# Supplementary material for: Altered Hippocampal-Prefrontal Neural Dynamics in Mouse Models of Down Syndrome
Source: Cell Rep. 2020 Jan 28;30(4):1152–1163.e4. doi: 10.1016/j.celrep.2019.12.065 (PMC6996020; doi:10.1016/j.celrep.2019.12.065)
Supplement: Document S1. Figures S1–S9 and Tables S1 and S3 [file mmc1.pdf]

Cell Reports, Volume *30*

Supplemental Information

Altered Hippocampal-Prefrontal Neural Dynamics in Mouse Models of Down Syndrome

Pishan Chang, Daniel Bush, Stephanie Schorge, Mark Good, Tara Canonica, Nathanael Shing, Suzanna Noy, Frances K. Wiseman, Neil Burgess, Victor L.J. Tybulewicz, Matthew C. Walker, and Elizabeth M.C. Fisher

Supplemental Information

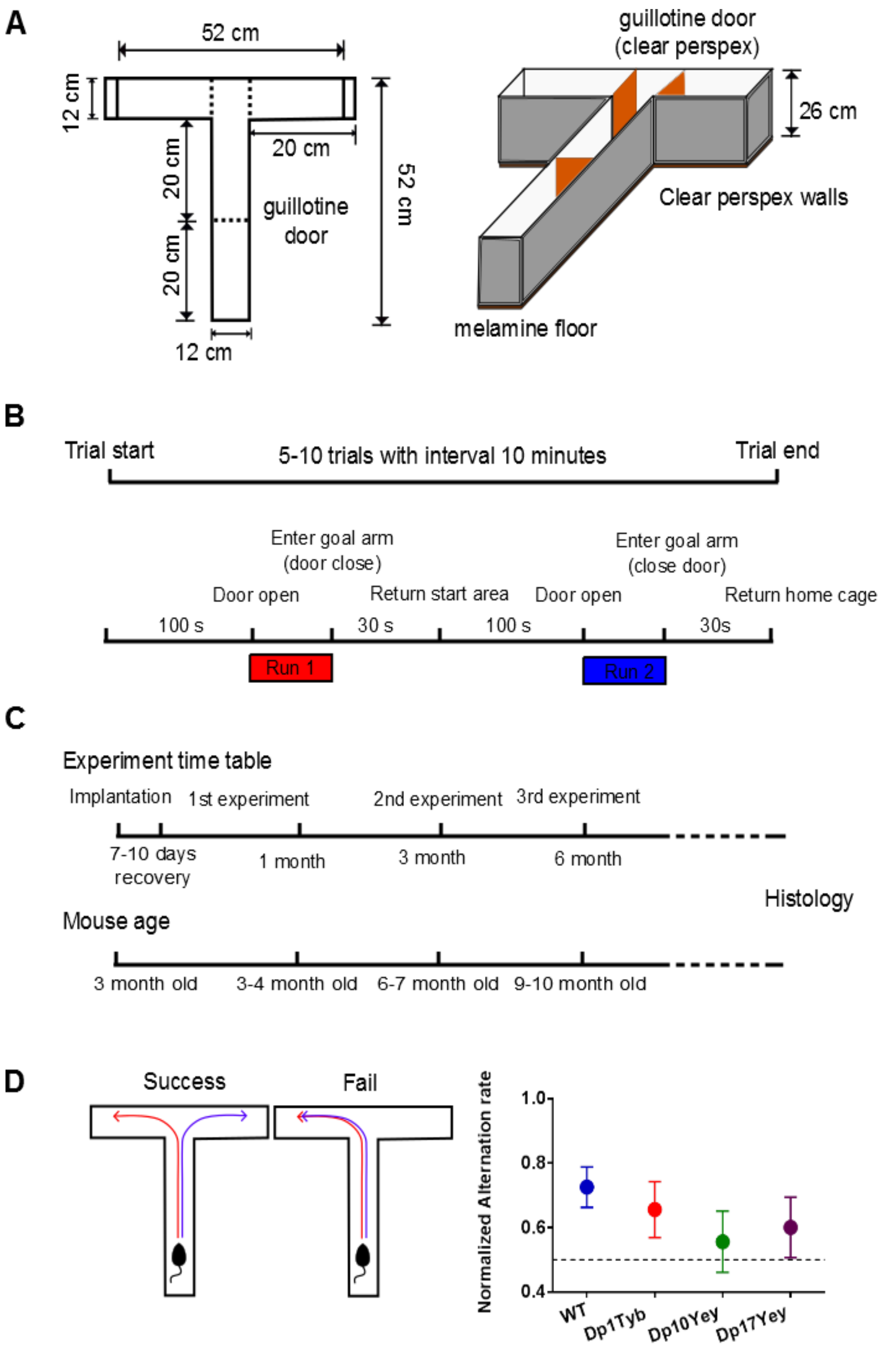

Figure S1: Further Details of the Behavioural Protocol (related to Figure 1)

Experimental protocol for the T-maze spontaneous alternation task, showing: **(A)** schematic of the T-maze; **(B)** trial protocol for probing spontaneous alternation behaviour maze; and **(C)** time line for the longitudinal study. **(D)** Schematic of successful alternation performance (left) alongside mean alternation performance for each mutant mouse group and pooled wild-type (WT) littermates (right, error bars showing 95% confidence intervals). This illustrates that alternation rate in the Dp10Yey animals is significantly different from WT and, importantly, not significantly greater than chance ($Z=-1.39$, $p=0.11$). Please refer to Supplemental Table 2 for full details of all statistical analyses.

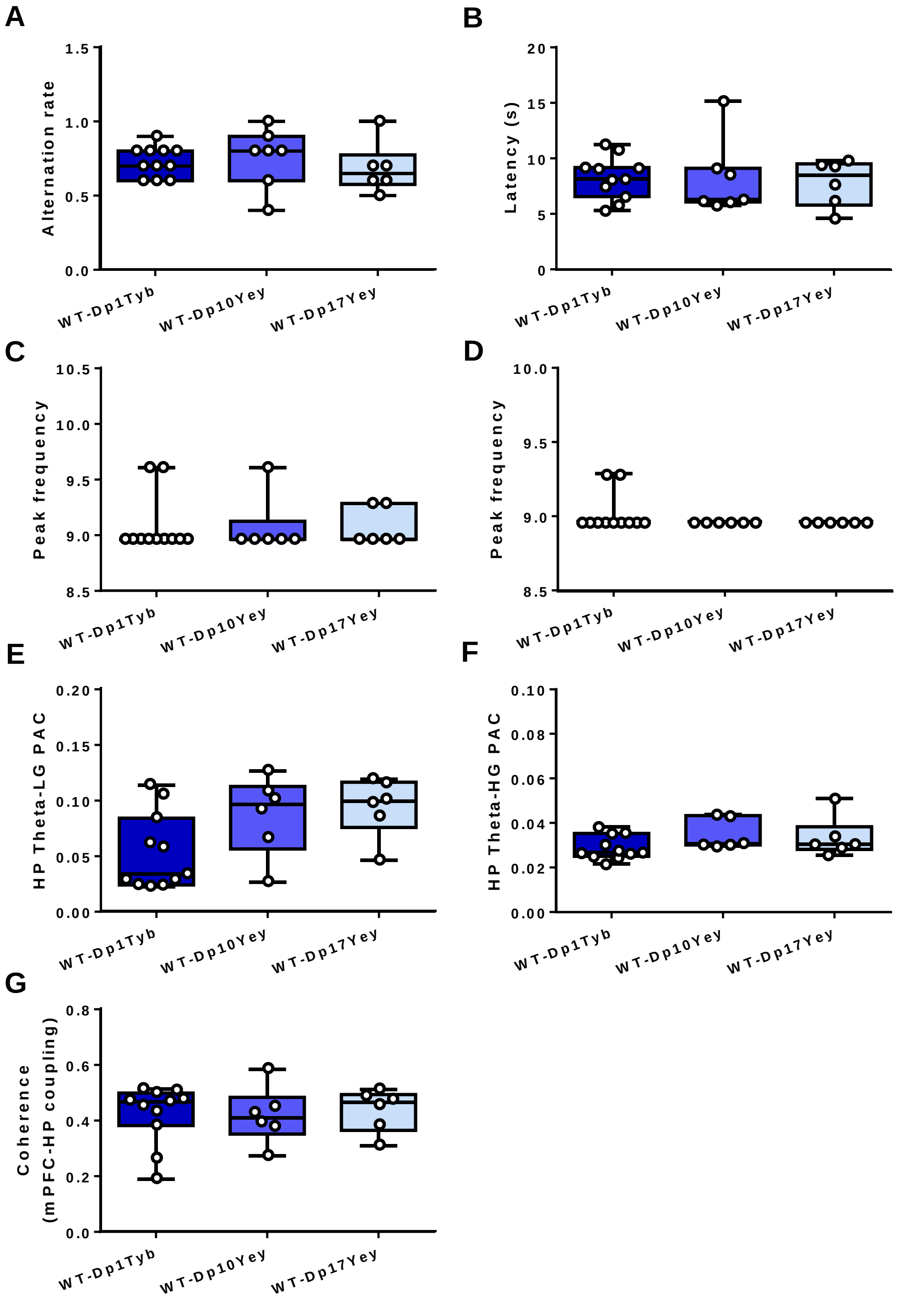

Figure S2: Comparison of Behavioural and Physiological Data across WT Groups (related to Figure 1)

Comparison of **(A)** spatial alternation rate; **(B)** trial latency; peak theta frequency in **(C)** hippocampus and **(D)** medial prefrontal cortex (mPFC); **(E)** theta-low gamma and **(F)** theta-high gamma phase-amplitude coupling in the hippocampus; and **(G)** theta coherence between hippocampus and mPFC across WT cohorts. Data are presented as box-whisker plots indicating the median, 25th and 75th percentiles, minimum and maximum values, with data for individual mice superimposed. There are no significant differences between WT groups in any panel. Please refer to Supplemental Table 2 for full details of all statistical analyses.

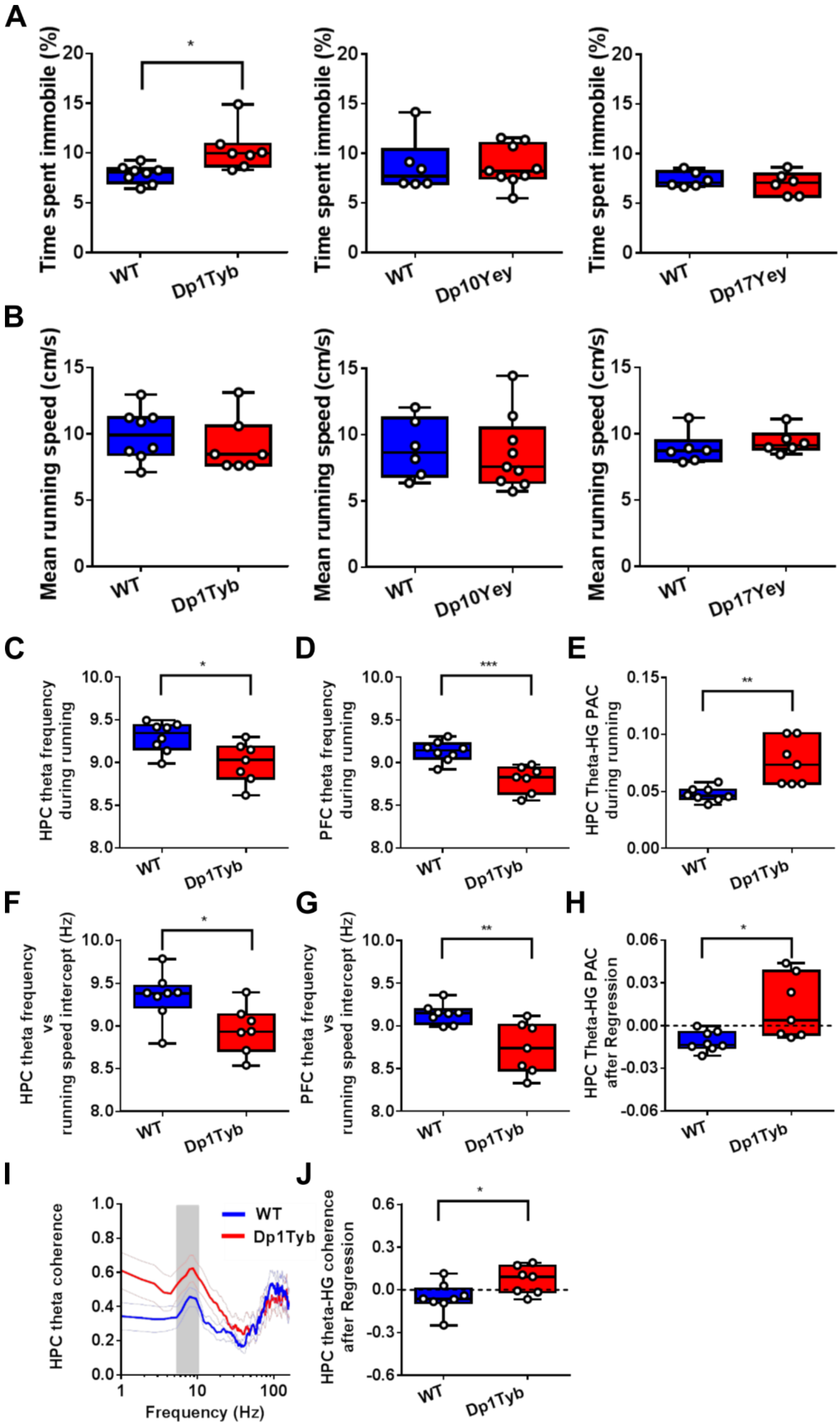

A
Time spent immobile (%)
WT
Dp1Tyb
Dp10Yey
Dp17Yey
B
Mean running speed (cm/s)
C
HPC theta frequency during running
D
PFC theta frequency during running
E
HPC Theta-HG PAC during running
F
HPC theta frequency vs running speed intercept (Hz)
G
PFC theta frequency vs running speed intercept (Hz)
H
HPC Theta-HG PAC after Regression
I
HPC theta coherence
Frequency (Hz)
J
HPC theta-HG coherence after Regression

Figure S3: Comparison of Movement Statistics across Groups (related to Figures 1 and 2)

(A) Relative period of each trial spent immobile (i.e. running speed <2cm/s); and **(B)** mean running speed during movement (i.e. running speed ≥2cm/s) across all mutant mouse and WT control groups, averaged across both runs. These data illustrate that the increased trial latency observed in Dp1Tyb mice results from significantly more time spent immobile ($t(13)=2.46$, $p<0.05$) without any difference in mean running speed during movement ($t(13)=-0.49$, $p=0.63$). No differences in time spent immobile or mean running speed during movement were observed in any other group compared to their WT cohort (all $p>0.38$), or between any of the WT groups (both $p>0.24$). **(C,D)** Average theta frequency and **(F,G)** intercept of the running speed v theta frequency relationship during movement for Dp1Tyb and WT groups in **(C,F)** hippocampus (HPC) and **(D,G)** medial prefrontal cortex (mPFC). Theta frequency during movement is significantly lower in Dp1Tyb animals in both HPC ($t(13)=-2.80$, $p<0.05$) and mPFC ($t(13)=-4.52$, $p<0.001$), due to a reduction in the intercept (HPC: $t(13)=-2.68$, $p<0.05$; mPFC: $t(13)=-3.45$, $p<0.01$) but not the slope (both $p>0.24$, data not shown) of the running speed v theta frequency relationship. **(E)** Average theta-HG PAC in HPC is significantly higher in Dp1Tyb animals when analyses are restricted to movement periods only ($t(13)=3.12$, $p<0.01$). **(H)** Moreover, the influence of average time immobile on theta-HG PAC is removed by linear regression across animals, the difference between groups is still significant ($t(13)=2.88$, $p<0.05$). **(I)** Average theta coherence between HPC and mPFC is significantly higher in Dp1Tyb animals when analyses are restricted to movement periods only ($t(13)=2.44$, $p<0.05$). **(J)** Moreover, if the influence of average time immobile on theta coherence is removed by linear regression across animals, the difference between groups is still significant ($t(13)=2.36$, $p<0.05$). Please refer to Supplemental Table 2 for full details of all statistical analyses.

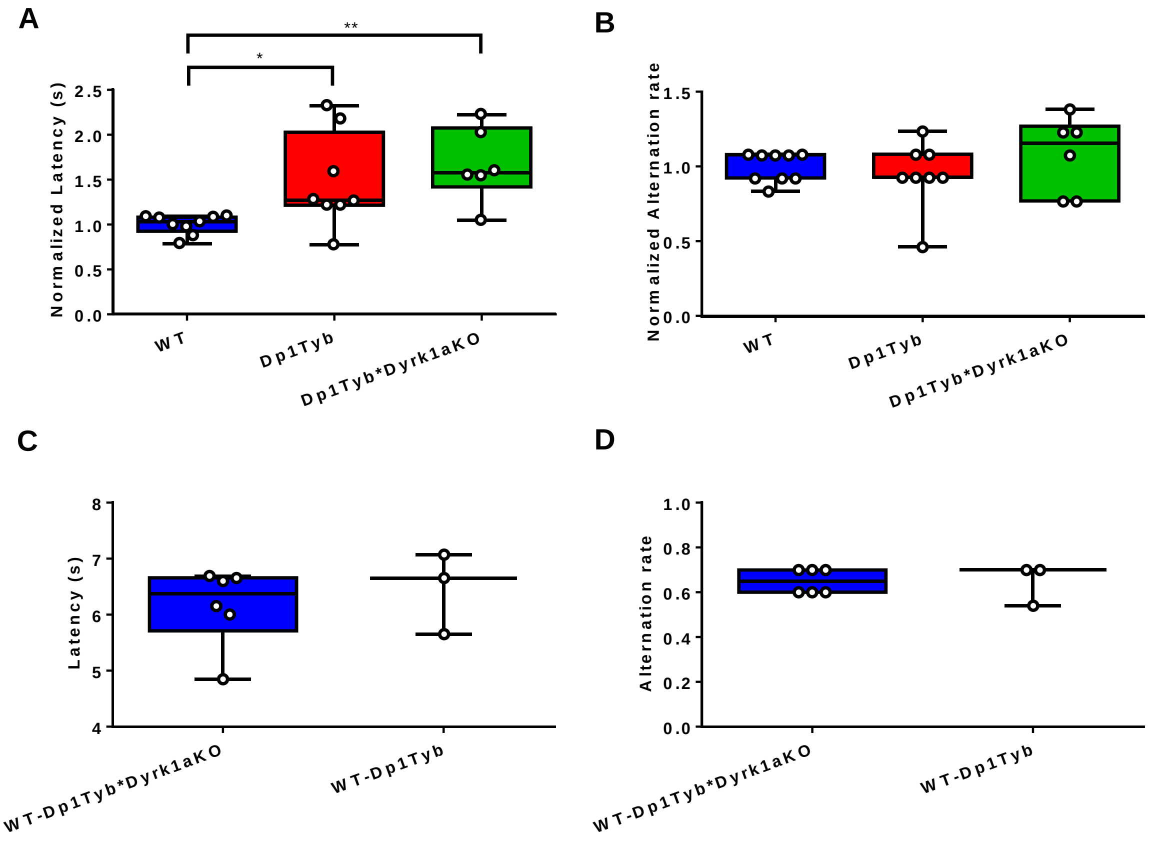

Figure S4: Behavioural Performance in Dp1Tyb and Dp1Tyb*Dyrk1aKO Mice (related to Figure 1)

(A) Trial latency and **(B)** alternation rate averaged over 10 trials for Dp1Tyb and Dp1Tyb*Dyrk1aKO mice compared to their pooled wild-type (WT) control group. All data are normalized to the WT mean. Trial latency differs significantly between groups (One way ANOVA, $F(2,20)=6.21$, $p<0.0001$). Post-hoc Tukey HSD indicates that trial latency is significant longer in Dp1Tyb and Dp1Tyb*Dyrk1aKO mice, compared to pooled WT ($p<0.05$ and $p<0.01$, respectively). **(C)** Comparison of trial latency and **(D)** alternation rate between WT cohorts, illustrating no significant differences in either case (Kruskal-Wallis test, both $p>0.58$). Data are presented as box-whisker plots indicating the median, 25th and 75th percentiles, minimum and maximum values, with data for individual mice superimposed. Please refer to Supplemental Table 2 for full details of all statistical analyses.

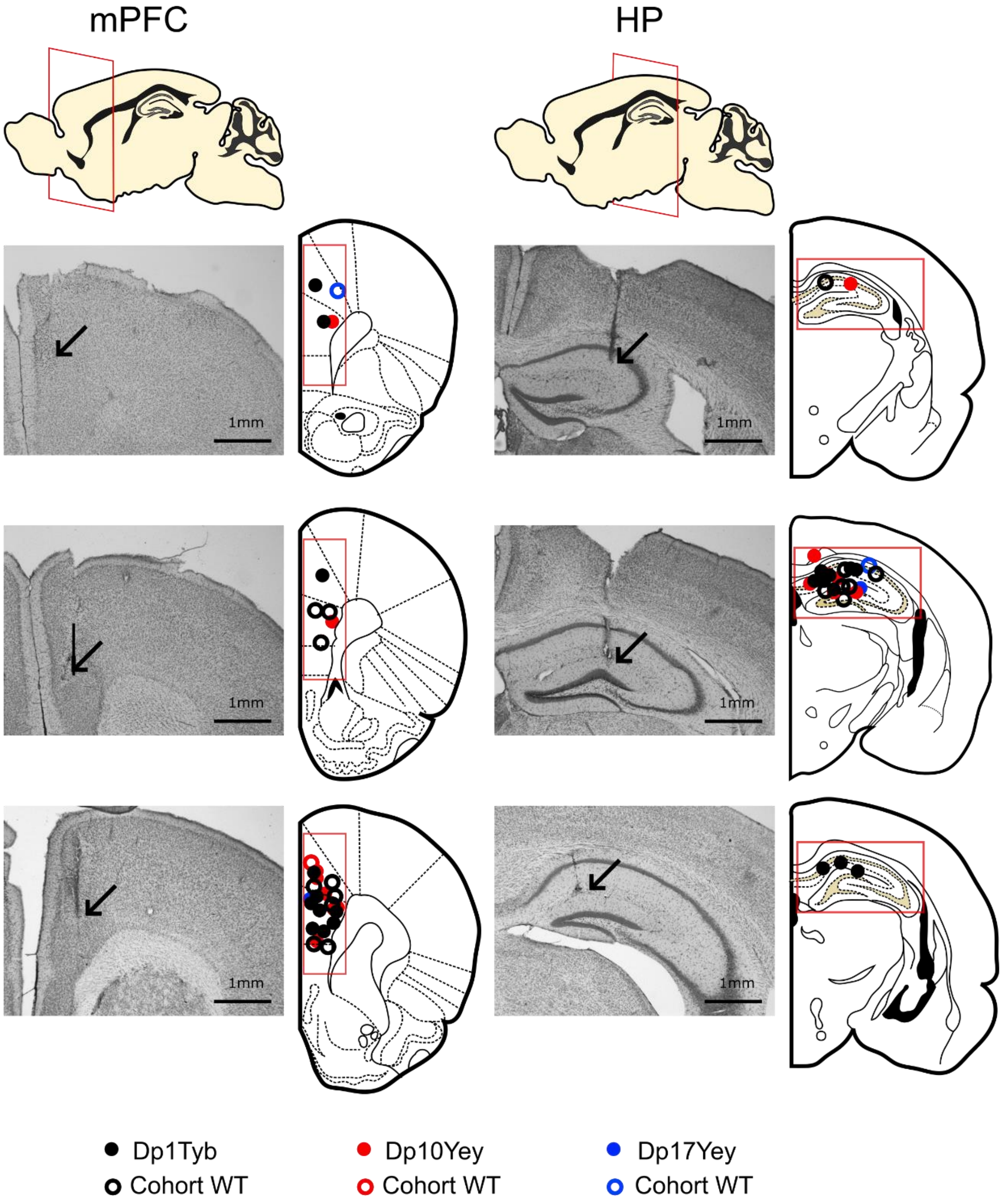

Figure S5: Histology (related to Figure 2)

Coronal sections of Nissl stained brains showing typical locations of recording electrode tips in medial prefrontal cortex and dorsal hippocampus. Arrows indicate the tip of each recording electrode. LFP data were only included in the study if electrode tips were located in mPFC (indicated by coloured rectangle) and dorsal hippocampus (HP, also indicated by coloured rectangle).

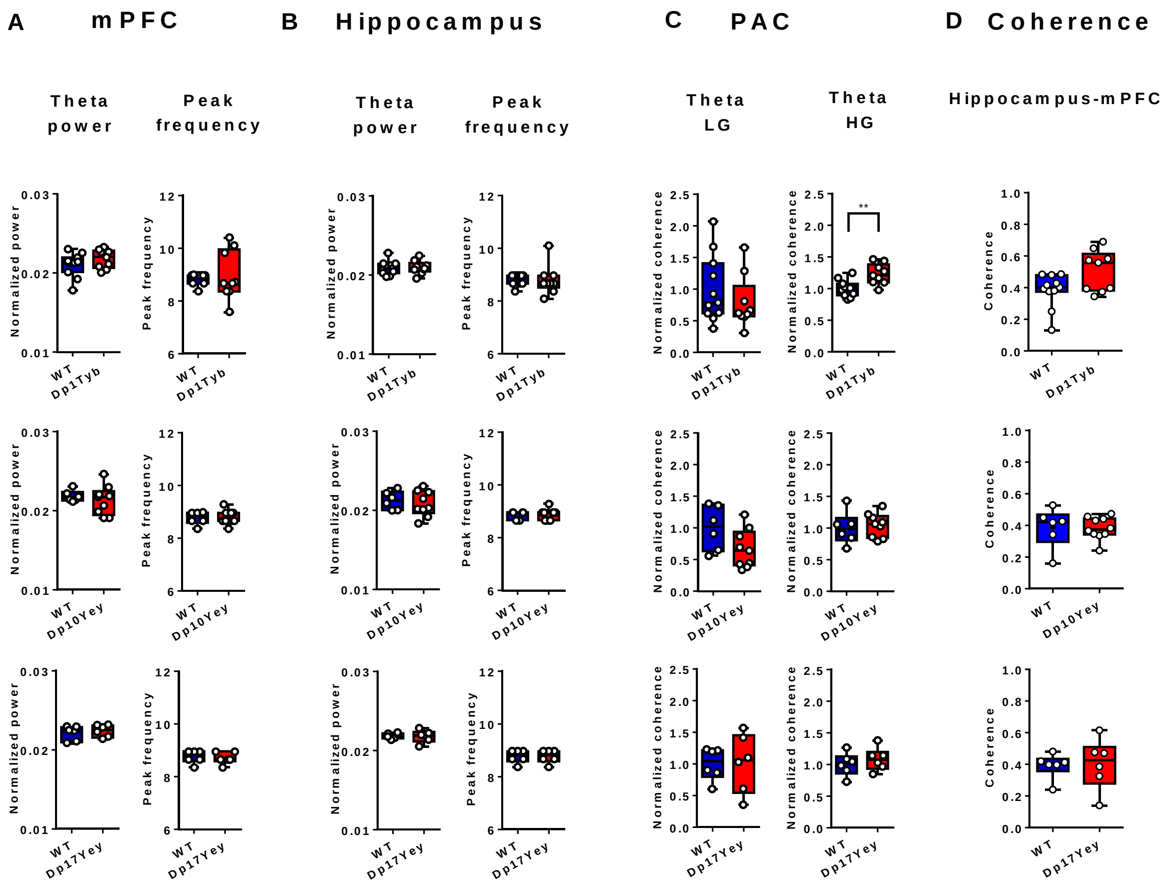

Figure S6: Habituation Phase Analyses (related to Figures 2, 3 and 4)

Comparison of average theta power and peak frequency in the **(A)** mPFC and **(B)** hippocampus, as well as **(C)** theta coherence between regions, across mutant and WT groups. There are no significant differences between any mutant mouse cohort and their WT control group in any of these parameters. Data are presented as box-whisker plots indicating the median, 25th and 75th percentiles, minimum and maximum values, with data for individual mice superimposed. Please refer to Supplemental Table 2 for full details of all statistical analyses.

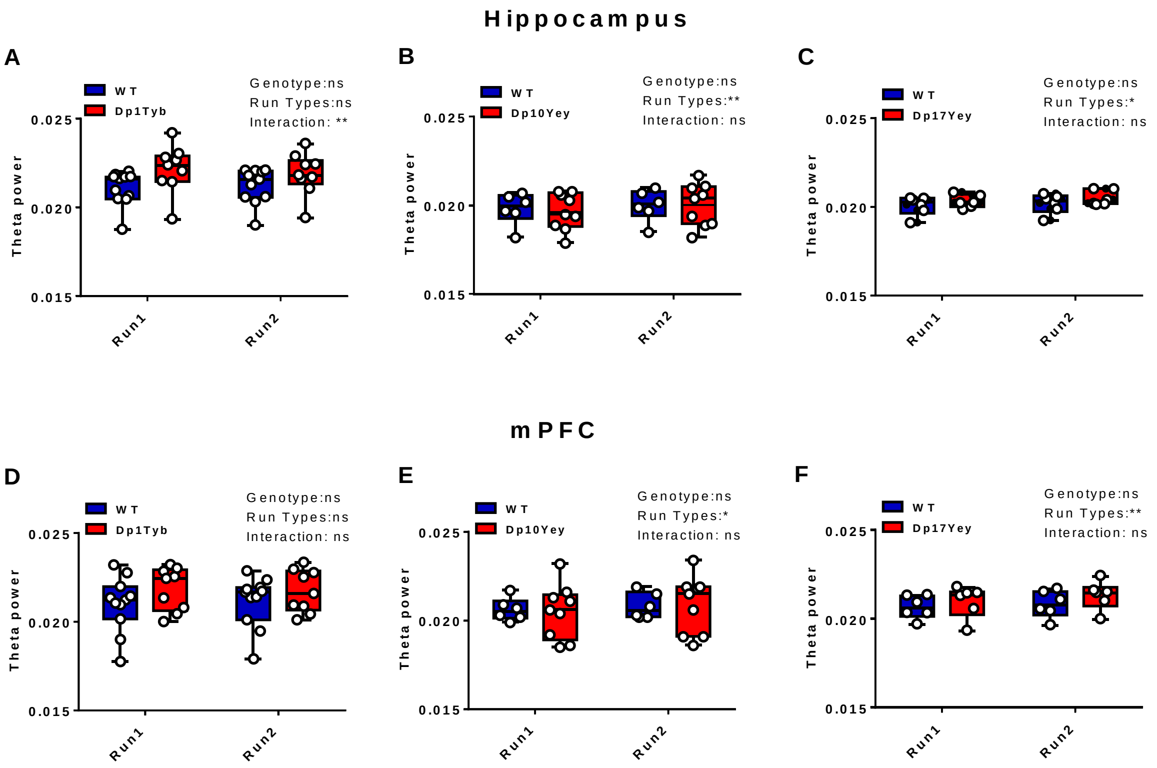

Figure S7: Comparison of Theta Power between Runs (related to Figure 2)

Average theta power during the first (sample) and second (choice) run on the T-maze in the **(A-C)** hippocampus and **(D-F)** mPFC in (A,D) Dp1Tyb and WT; (B,E) Dp10Yey and WT; and (C,F) Dp17Yey and WT animals. Two-way ANOVAs with levels of run (first v second) and genotype (mutant v WT) show a main effect of run on hippocampal theta power in the Dp10Yey and WT ($F(1,14)=6.95$, $p<0.05$), Dp17Yey and WT ($F(1,10)=8.35$, $p<0.05$) cohorts; and a run x genotype interaction in the Dp1Tyb and WT cohort ($F(1,18)=8.86$, $p<0.05$). The same analysis also reveals a main effect of run on mPFC theta power in the Dp10Yey and WT ($F(1,14)=7.67$, $p<0.05$), Dp17Yey and WT ($F(1,10)=17.56$, $p<0.05$) cohorts; but no run x genotype interaction in the Dp1Tyb and WT cohort in this case ($F(1,18)=2.88$, $p>0.05$). Data are presented as box-whisker plots indicating the median, 25th and 75th percentiles, minimum and maximum values, with data for individual mice superimposed. Please refer to Supplemental Table 2 for full details of all statistical analyses.

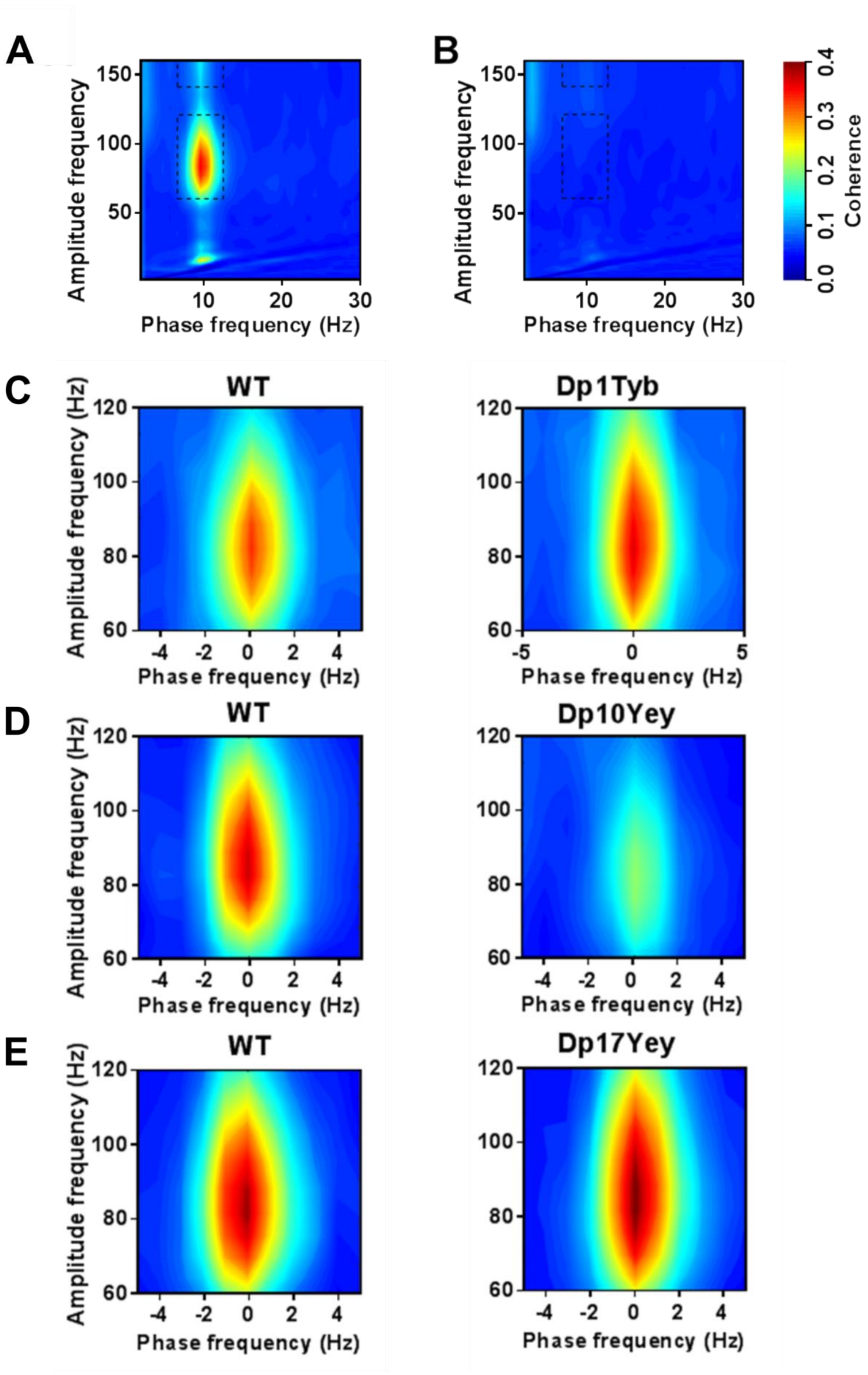
A
B
Amplitude frequency
Phase frequency (Hz)
Coherence
C
WT
Dp1Tyb
Amplitude frequency (Hz)
Phase frequency (Hz)
D
WT
Dp10Yey
E
WT
Dp17Yey

Figure S8: Phase-amplitude Comodulograms (related to Figure 3)

(A, B) Phase-amplitude comodulograms, averaged across all DS model and WT control groups at 3 months of age for **(A)** hippocampus and **(B)** mPFC. Visual inspection reveals strong peaks between 6-12Hz theta phase and both 60-120Hz 'low gamma' (LG) and 140-160Hz 'high gamma' (HG) rhythms in the hippocampus, with no strong phase-amplitude coupling apparent in the mPFC (see Figure 3 for details of comparisons between groups). **(C-E)** Comodulograms showing hippocampal phase-amplitude coupling between 6-12Hz theta phase and 60-120Hz 'low gamma' (LG) amplitude separately for the **(C)** Dp1Tyb and WT; **(D)** Dp10Yey and WT; **(E)** Dp17Yey and WT groups. Theta phase frequencies have been aligned to the peak theta frequency in each animal, to facilitate comparison, and colour axes have been matched across groups to illustrate the relative strength of theta-LG phase-amplitude coupling.

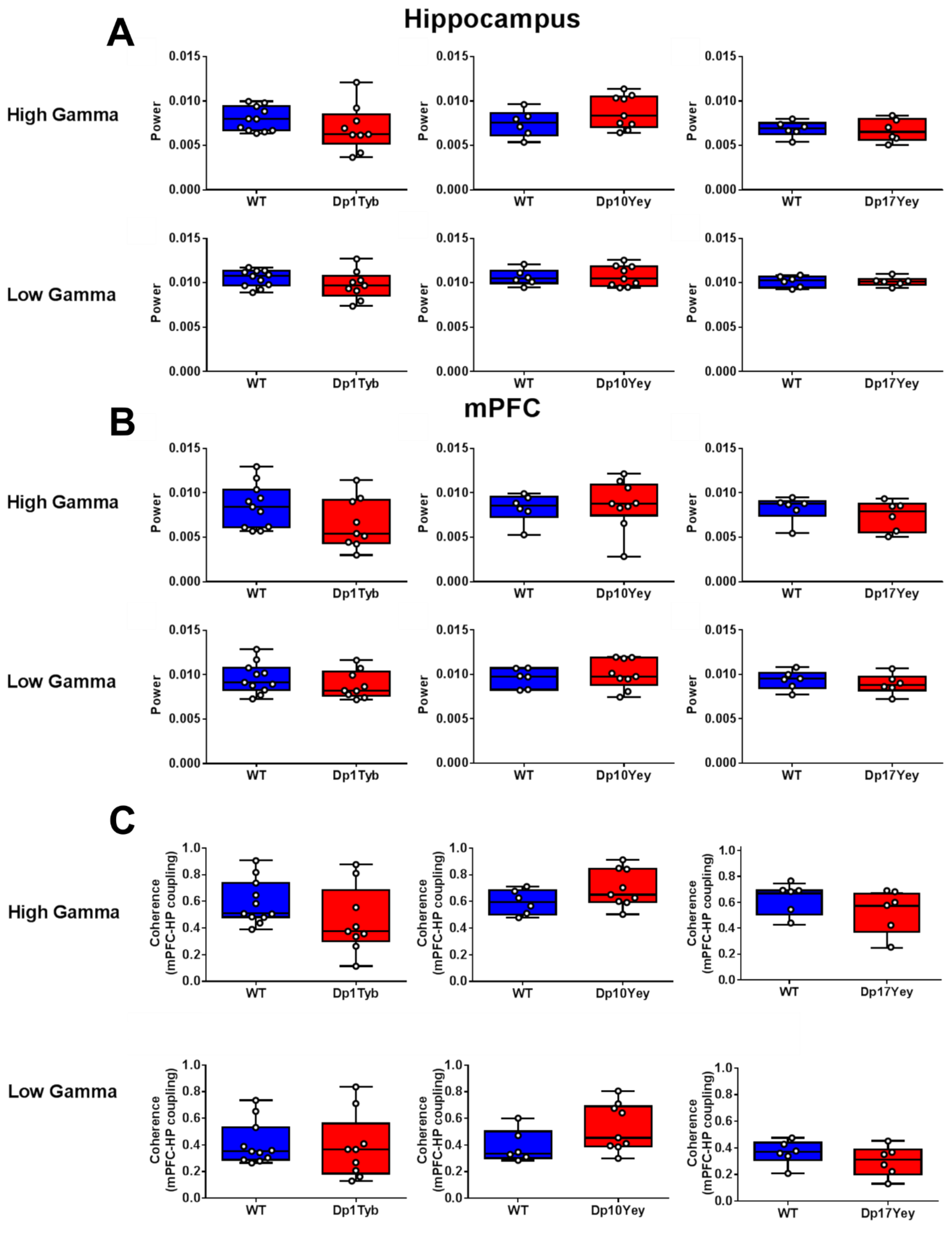
A
Hippocampus
High Gamma
Low Gamma
Power
0.015
0.010
0.005
0.000
WT
Dp1Tyb
Dp10Yey
Dp17Yey
B
mPFC
High Gamma
Low Gamma
C
Coherence (mPFC-HP coupling)
1.0
0.8
0.6
0.4
0.2
0.0

Figure S9: Gamma Power and Coherence across Groups (related to Figures 2 and 4)

(A,B) Average power in the low (60-120Hz) and high (120-140Hz) gamma bands in **(A)** hippocampus and **(B)** mPFC for each mutant mouse and control group during spontaneous alternation on the T-maze, which show no significant differences in any instance. **(C)** Coherence between the hippocampus (HP) and mPFC in the high- and low- gamma frequency bands for each mutant mouse and WT control group, which show no significant differences in any instance. Data are presented as box-whisker plots indicating the median, 25th and 75th percentiles, minimum and maximum values, with data for individual mice superimposed. Please refer to Supplemental Table 2 for full details of all statistical analyses.

Table S1: Summary of Trial and Animal Numbers at 3 months of Age

Related to Figures 1-4

	Summary						
	**Dp1Tyb**	**WT**	**Dp10Yey**	**WT**	**Dp17Yey**	**WT**	**Mean**
**n**	9	11	10	6	6	6	8
**All Trials (Mean)**	9.33	8.64	8.70	7.83	10.17	9.83	9.08
**All Trials (SD)**	2.00	2.38	2.11	2.79	0.41	0.41	1.68
**Artefact Trials (Mean)**	2.00	1.73	1.80	0.50	1.00	0.33	1.23
**Artefact Trials (SD)**	2.74	2.33	2.66	1.22	0.89	0.82	1.78
**Trials Included (Mean)**	7.33	6.91	6.90	7.33	9.17	9.50	7.86
**Trials Included (SD)**	2.69	2.30	3.63	2.58	0.98	0.84	2.17

Table S3: Summary of Trial and Animal numbers for the Longitudinal Study

Related to Figure 5

3 months							
	**Dp1Tyb**	**WT**	**Dp10Yey**	**WT**	**Dp17Yey**	**WT**	**Mean**
**n**	4	6	5	3	6	5	4.83
**All Trials (Mean)**	8.75	8.17	9.40	6.67	10.17	9.80	8.83
**All Trials (SD)**	1.89	2.48	0.89	3.51	0.41	0.45	1.61
**Artefact Trials (Mean)**	2.50	1.17	2.00	0.00	1.00	0.40	1.18
**Artefact Trials (SD)**	3.32	2.40	3.39	0.00	0.89	0.89	1.82
**Included Trials (Mean)**	6.25	7.00	7.40	6.67	9.17	9.40	7.65
**Included Trials (SD)**	2.87	2.61	4.22	3.51	0.98	0.89	2.51

6 months							
	**Dp1Tyb**	**WT**	**Dp10Yey**	**WT**	**Dp17Yey**	**WT**	**Mean**
**n**	4	6	5	3	6	5	4.83
**All Trials (Mean)**	12.50	10.83	10.00	10.33	10.00	10.00	10.61
**All Trials (SD)**	4.36	1.17	0.00	0.58	0.00	0.00	1.02
**Artefact Trials (Mean)**	0.00	0.33	0.00	0.00	0.67	0.20	0.20
**Artefact Trials (SD)**	0.00	0.52	0.00	0.00	0.82	0.45	0.30
**Included Trials (Mean)**	12.50	10.50	10.00	10.33	9.33	9.80	10.41
**Included Trials (SD)**	4.36	1.52	0.00	0.58	0.82	0.45	1.29

9 months							
	**Dp1Tyb**	**WT**	**Dp10Yey**	**WT**	**Dp17Yey**	**WT**	**Mean**
**n**	3	4	4	2	6	5	4
**All Trials (Mean)**	10.33	12.25	10.25	10.00	10.17	10.40	10.57
**All Trials (SD)**	0.58	5.19	2.06	0.00	0.41	0.55	1.46
**Artefact Trials (Mean)**	0.00	0.25	0.00	0.50	0.33	0.00	0.18
**Artefact Trials (SD)**	0.00	0.50	0.00	0.71	0.52	0.00	0.29
**Included Trials (Mean)**	10.33	12.00	10.25	9.50	9.83	10.40	10.39
**Included Trials (SD)**	0.58	4.69	2.06	0.71	0.75	0.55	1.56
