# Supplementary material for: Altered Hippocampal-Prefrontal Neural Dynamics in Mouse Models of Down Syndrome
Source: Cell Rep. 2020 Jan 28;30(4):1152–1163.e4. doi: 10.1016/j.celrep.2019.12.065 (PMC6996020; doi:10.1016/j.celrep.2019.12.065)
Supplement: Document S2. Article plus Supplemental Information [file mmc3.pdf]

Cell Reports

Article

Altered Hippocampal-Prefrontal Neural Dynamics in Mouse Models of Down Syndrome

Graphical Abstract

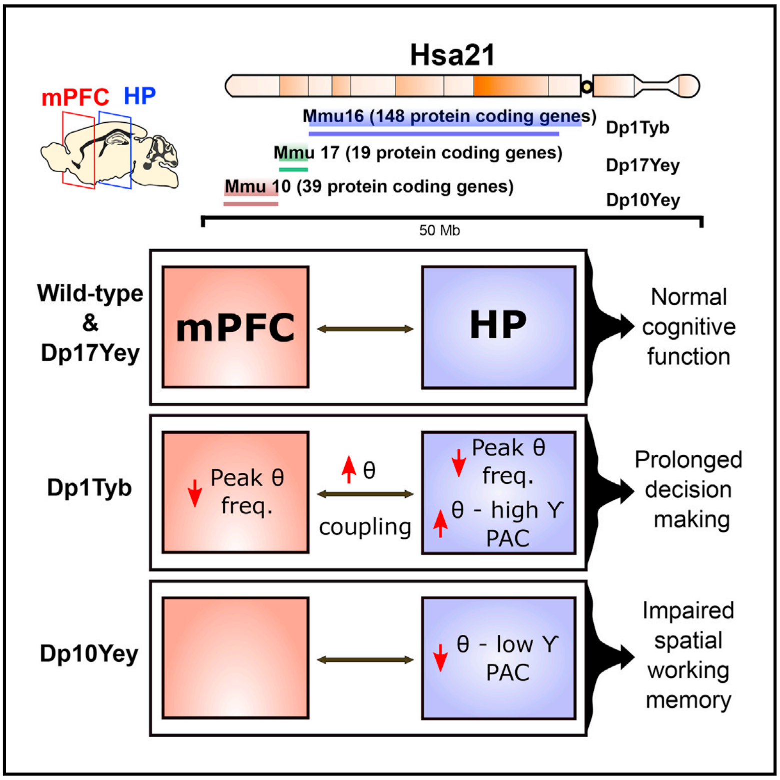

Highlights

- Study of behavior and EEG in three partially trisomic mouse models of Down syndrome (DS)
- These mutant mice show non-overlapping differences in spatial working memory function
- Behavioral changes segregate with distinct EEG abnormalities in the hippocampus and mPFC
- This links cognitive deficits to specific changes in hippocampal and mPFC circuit dynamics

Authors

Pishan Chang, Daniel Bush, Stephanie Schorge, ..., Victor L.J. Tybulewicz, Matthew C. Walker, Elizabeth M.C. Fisher

Correspondence

m.walker@ucl.ac.uk

In Brief

Chang et al. examine three partially trisomic mouse models of Down syndrome that together cover all regions of homology with human chromosome 21. They identify non-overlapping differences in spatial working memory function associated with distinct abnormalities in hippocampal and medial prefrontal electrophysiology.

Chang et al., 2020, Cell Reports *30*, 1152–1163
January 28, 2020 © 2019 The Author(s).
https://doi.org/10.1016/j.celrep.2019.12.065

CellPress

OPEN ACCESS
CellPress

Cell Reports
Article

Altered Hippocampal-Prefrontal Neural Dynamics in Mouse Models of Down Syndrome

Pishan Chang,[1,2] Daniel Bush,[3] Stephanie Schorge,[1] Mark Good,[4] Tara Canonica,[4] Nathanael Shing,[1] Suzanna Noy,[2] Frances K. Wiseman,[2] Neil Burgess,[3] Victor L.J. Tybulewicz,[5,6,7] Matthew C. Walker,[1,7,8,*] and Elizabeth M.C. Fisher[2,7]

[1]Department of Clinical and Experimental Epilepsy, UCL Queen Square Institute of Neurology, London WC1N 3BG, UK
[2]Department of Neuromuscular Diseases, UCL Queen Square Institute of Neurology, London WC1N 3BG, UK
[3]UCL Institute of Cognitive Neuroscience, UCL Queen Square Institute of Neurology, University College London WC1N 3AZ, UK
[4]School of Psychology, College of Biomedical and Life Sciences, Cardiff University, Cardiff CF10 3AT, UK
[5]Francis Crick Institute, London NW1 1AT, UK
[6]Department of Medicine, Imperial College, London W12 0NN, UK
[7]These authors contributed equally
[8]Lead Contact
*Correspondence: m.walker@ucl.ac.uk
https://doi.org/10.1016/j.celrep.2019.12.065

SUMMARY

Altered neural dynamics in the medial prefrontal cortex (mPFC) and hippocampus may contribute to cognitive impairments in the complex chromosomal disorder Down syndrome (DS). Here, we demonstrate non-overlapping behavioral differences associated with distinct abnormalities in hippocampal and mPFC electrophysiology during a canonical spatial working memory task in three partially trisomic mouse models of DS (Dp1Tyb, Dp10Yey, and Dp17Yey) that together cover all regions of homology with human chromosome 21 (Hsa21). Dp1Tyb mice show slower decision-making (unrelated to the gene dose of *DYRK1A*, which has been implicated in DS cognitive dysfunction) and altered theta dynamics (reduced frequency, increased hippocampal-mPFC coherence, and increased modulation of hippocampal high gamma); Dp10Yey mice show impaired alternation performance and reduced theta modulation of hippocampal low gamma; and Dp17Yey mice are not significantly different from the wild type. These results link specific hippocampal and mPFC circuit dysfunctions to cognitive deficits in DS models and, importantly, map them to discrete regions of Hsa21.

INTRODUCTION

Down syndrome (DS) is a complex cognitive disorder arising from the trisomy of human chromosome 21 (Hsa21), with an incidence of ~1 in 800 live births worldwide (de Graaf et al., 2015). The current global population of people with DS is estimated at 6 million (Hanney et al., 2012), and prevalence is rising, primarily due to an increase in maternal age (a major risk factor for DS) and increased life expectancy in people with DS, resulting from reduced infant mortality rates and improved access to health-care (Loane et al., 2013; Sherman et al., 2007; Wu and Morris, 2013). DS is characterized by intellectual disability (Grieco et al., 2015; Lott and Dierssen, 2010) and prominent impairments in planning, decision-making, and memory function (Clark et al., 2017; Grieco et al., 2015; Lanfranchi et al., 2010; Lavenex et al., 2015; Pennington et al., 2003; Rowe et al., 2006), which likely arise from functional abnormalities of the hippocampus and medial prefrontal cortex (mPFC; Anderson et al., 2013; Lott and Dierssen, 2010; Nadel, 2003; Nelson et al., 2005; Pennington et al., 2003; Rowe et al., 2006; Ruiz-Mejias et al., 2016). Increased dosages of single genes in Hsa21, such as *Dyrk1A*, have been proposed to account for many of the alterations in neural development and abnormal phenotypes associated with DS and are thus targets for therapy development (Duchon and Herault, 2016).

Activity in the hippocampus and mPFC can be characterized by oscillations in the theta and gamma bands. Hippocampal theta oscillations are associated with translational movement (Bohbot et al., 2017; O'Keefe and Nadel, 1978) and mnemonic function (Fell and Axmacher, 2011; Guderian et al., 2009; Winson, 1978) across species and can modulate synaptic plasticity (Hasselmo et al., 2002). Moreover, hippocampal theta modulates the amplitude of concomitant gamma oscillations both locally and across the neocortex (Buzsáki and Chrobak, 1995; Canolty et al., 2006; Sirota et al., 2008), and task-related increases in phase-amplitude coupling are associated with successful memory encoding (Tort et al., 2009). In humans, theta oscillations in the mPFC are observed during working memory maintenance (Griesmayr et al., 2010; Raghavachari et al., 2001) and long-term memory retrieval (Kaplan et al., 2014; Klimesch et al., 2001), while increases in theta coherence between the hippocampus and mPFC are associated with planning and decision-making across species (Benchenane et al., 2010; Guitart-Masip et al., 2013; Jones and Wilson, 2005; Siapas et al., 2005; Young and McNaughton, 2009).

To further elucidate the neural mechanisms underlying cognitive deficits associated with DS, we studied three chromosome-engineered mouse models that each exhibit trisomy for one region of orthology with human chromosome Hsa21: the Dp1Tyb, Dp10Yey, and Dp17Yey strains (full nomenclature given

 © 2019 The Author(s).
This is an open access article under the CC BY license (http://creativecommons.org/licenses/by/4.0/).

in STAR Methods; Lana-Elola et al., 2016; Yu et al., 2010a). In combination, these three mouse strains are triplicated for almost all the genes on Hsa21. We hypothesized that these trisomic mice might exhibit distinct cognitive impairments, corresponding to distinct alterations in oscillatory activity patterns within the hippocampus and mPFC (Anderson et al., 2013; Ruiz-Mejias et al., 2016). Hence, we carried out simultaneous local field potential (LFP) recordings from those regions while mice performed a canonical test of spatial working memory—the spontaneous alternation task—which, importantly, can dissociate mnemonic function (i.e., alternation success; Deacon and Rawlins, 2006; Sarnyai et al., 2000; Wenk, 2001) from planning and decision-making processes (i.e., trial latency; Bizon et al., 2012; Pioli et al., 2014).

Here, we demonstrate that distinct behavioral impairments associated with DS are exhibited by animals with different regions of homology and, crucially, that these impairments are associated with distinct alterations in neural dynamics across the hippocampus and mPFC. Moreover, reducing the "dose" of *Dyrk1a*—a gene that has been suggested to be critically important for neural function in DS (Arron et al., 2006; Fotaki et al., 2002; Guimera et al., 1999; Hämmerle et al., 2008; Park et al., 2009; Tejedor and Hämmerle, 2011)—was not sufficient to rescue the observed differences in behavior, supporting the hypothesis that cognitive impairments in DS do not necessarily map to single genes. By taking an unbiased approach to the gene content of these partially trisomic mice, and by combining behavioral and electrophysiological methodologies, we have therefore identified a critical circuit dysfunction in DS models that paves the way for the future determination of key dosage-sensitive gene combinations underlying cognitive phenotypes in this complex chromosomal disorder.

RESULTS

Impaired Spatial Working Memory in Dp10Yey Mice and Decision-Making in Dp1Tyb Mice

Impairments in planning, decision-making, and memory function have a significant impact on the lives of people with DS. In order to dissect the mechanisms underlying these cognitive deficits, we studied three mouse lines that are triplicated for the three mouse chromosome regions syntenic to Hsa21. The Dp1Tyb mouse strain has a 23-Mb duplication of the Hsa21-syntenic region of Mmu16, which contains 148 coding genes with orthologs on Hsa21 (Lana-Elola et al., 2016); the Dp10Yey strain is duplicated for the Hsa21-syntenic region of Mmu10, which encodes 39 Hsa21 protein-coding genes; and the Dp17Yey line is duplicated for the Hsa21-syntenic region of Mmu17, which encodes 19 protein-coding genes (Yu et al., 2010a). Together, these mice make up a "mapping panel," such that phenotypes found in any one strain are likely to arise from having an additional (i.e., third) copy of the specific Hsa21 orthologs within that strain.

We began by comparing cognitive function in male Dp1Tyb, Dp17Yey, and Dp10Yey mice at 3 months of age with age- and sex-matched wild-type (WT) littermate control cohorts using a canonical spatial alternation task (Figures 1A–1C; see Figures S1A–S1C for further details and Table S1 for trial and animal numbers). Importantly, this task can assay both mnemonic function (by examining the propensity to spontaneously alternate between goal arms on successive trials) and decision-making (by examining the time taken to choose and enter a goal arm). Intriguingly, we found that distinct functional deficits were exhibited by each mutant mouse strain, suggesting that the trisomy of discrete Hsa21 orthologs can have divergent effects on cognitive function.

First, we found that Dp10Yey mice exhibited alternation rates that were significantly lower than their WT littermates and did not differ from chance (Figure 1E). In contrast, alternation rates in Dp1Tyb and Dp17Yey mice did not differ significantly from those of WT mice and were significantly above chance in both strains (Figures 1D and 1F; see Figure S1D for further details), with no difference in alternation rates between WT cohorts (Figure S2A). These results suggest that Dp10Yey mice have impaired memory function.

Second, we examined trial latencies, defined as the time taken to make a final crossing of the decision point prior to turning into the goal arm (see STAR Methods). We found that these were significantly greater in Dp1Tyb mice compared to their WT littermates (Figure 1G), while no differences were observed between Dp10Yey or Dp17Yey mice and their respective control groups (Figures 1H and 1I) or among any of the WT cohorts (Figure S2B). Importantly, the increased trial latency exhibited by Dp1Tyb mice could not simply be accounted for by motor impairments, independent of decision-making processes, as we found no differences in average running speed between any mutant mouse group and their WT littermates. Conversely, Dp1Tyb mice spent a significantly greater proportion of each trial immobile, prior to making a decision, with no differences between either of the other mutant mouse strains and their control groups (Figure S3). In sum, these results suggest that decision-making processes are disrupted in Dp1Tyb mice, despite relatively intact mnemonic function, while Dp10Yey and Dp17Yey mice are spared.

Finally, previous studies of transgenic mouse models of DS have led to the proposal that the overexpression of *Dyrk1a* (and thus an increased dosage of the DYRK1A protein) makes a critical contribution to neurological and behavioral abnormalities by shifting the excitation/inhibition balance toward inhibition, for example (Ruiz-Mejias et al., 2016; Souchet et al., 2014). The *Dyrk1a* gene maps to the Mmu16 region of Hsa21 and so is duplicated within the Dp1Tyb strain. To assess the behavioral consequences of altering the copy number of *Dyrk1a* in Dp1Tyb mice, we crossed Dp1Tyb animals with those carrying a disrupted *Dyrk1a* gene to generate Dp1Tyb*Dyrk1aKO mice that are still duplicated for 147 Hsa21-orthologous coding genes on Mmu16, but have only two functional copies of *Dyrk1a*. Interestingly, these Dp1Tyb*Dyrk1aKO mice exhibited both a similar alternation rate to Dp1Tyb mice and a similarly prolonged decision-making (latency) phenotype (Figure S4). Thus, the reduction of the *Dyrk1a* copy number from three to two did not rescue the increased trial latency exhibited by Dp1Tyb mice. This finding indicates that the triplication of *Dyrk1a* is not necessary to produce the decision-making deficit in Dp1Tyb mice, which must therefore arise from other gene(s) in this region of Hsa21 homology.

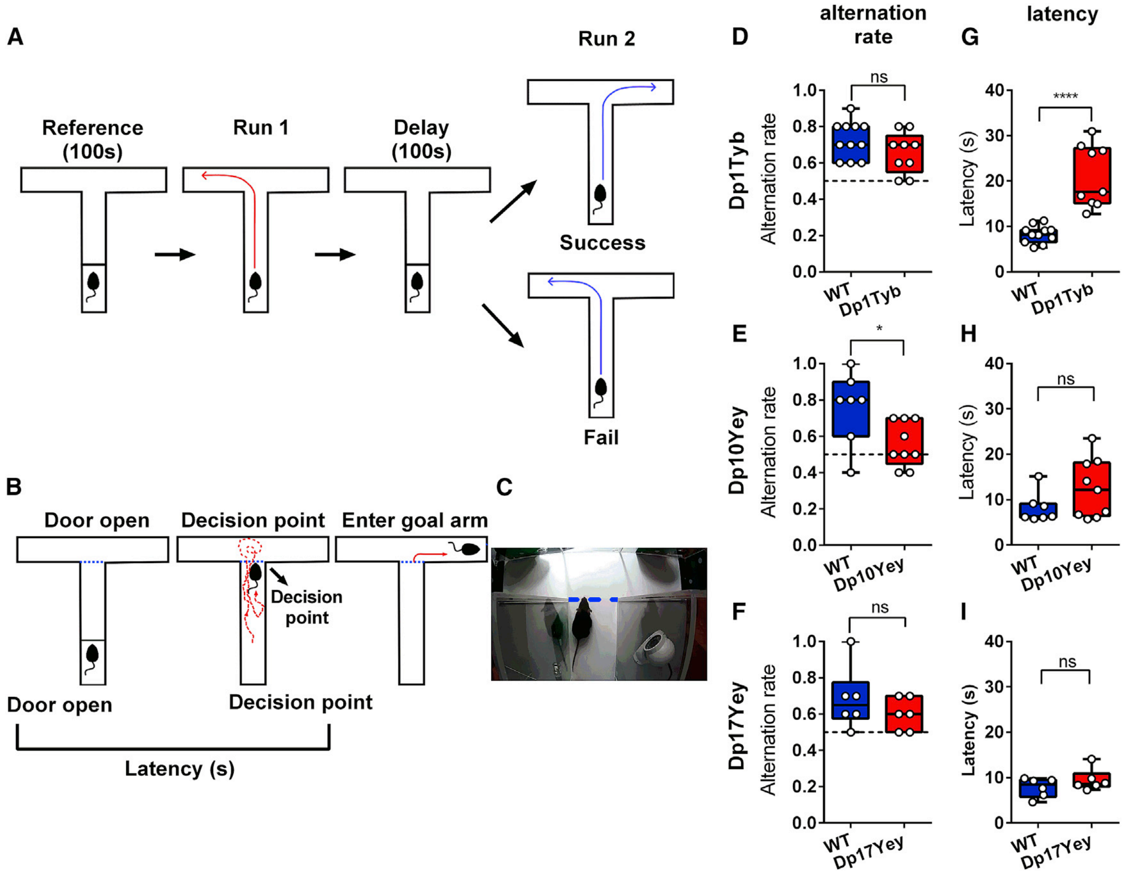

Figure 1. Spatial Alternation Rate and Trial Latency in Mouse Models of DS

(A) Schematic experimental procedure (see Figures S1A–S1C for further details).

(B) Schematic method for computing trial latency: the time between raising the door to release the animal from the start area to the point at which the mouse's nose crosses the "decision point" (blue dashed line) before entering a goal arm.

(C) Example of one animal reaching the decision point.

(D–I) Alternation rate (D–F) and trial latency (G–I) for each DS mouse model compared to their wild-type (WT) control group, showing (E) significant differences in alternation rate for Dp10Yey versus WT mice ($t(14) = 2.48$, $p < 0.05$) and (G) significant differences in trial latency for Dp1Tyb versus WT mice ($t(18) = 5.97$, $p < 0.001$), but no differences in either measure for Dp17Yey versus WT mice. Chance alternation rate is shown as a black dotted line.

Data are presented as box-whisker plots indicating the median, 25th and 75th percentiles, and minimum and maximum values, with data for individual mice superimposed.

Please refer to Table S1 for trial and animal numbers and Table S2 for full details of all statistical analyses.

Reduced Theta Frequency in Dp1Tyb Mice

Successful memory encoding and retrieval are associated with increased theta power in both the hippocampus (Fell and Axmacher, 2011; Guderian et al., 2009; Winson, 1978) and mPFC (Griesmayr et al., 2010; Kaplan et al., 2014; Klimesch et al., 2001; Raghavachari et al., 2001) across species. Furthermore, a reduction in hippocampal theta frequency has been directly linked to impaired spatial memory performance in a rodent model of temporal lobe epilepsy (Richard et al., 2013). Hence, we next analyzed LFP recordings from the hippocampus and mPFC during spatial alternation in the T-maze (see Figure S5 for details of electrode placement). Initially, we focused our analyses on a 10-s window centered on the time at which animals crossed the decision point, averaged across periods of memory encoding and retrieval from the sample and choice runs, respectively (see STAR Methods for further details).

As expected, the average power spectra from the mPFC (Figures 2A–2C) and hippocampus (Figures 2D–2F) across all animals showed a prominent peak in the 6–12-Hz theta band during this period. Interestingly, although theta power did not differ between mouse lines, we found that theta frequency in both the mPFC (Figure 2A) and hippocampus (Figure 2D) was consistently lower in Dp1Tyb mice than in WT controls. In contrast, no difference in theta frequency was observed in either region in Dp10Yey or Dp17Yey mice, compared to their control cohorts (Figures 2B, 2C, 2E and 2F), or between WT cohorts (Figures S2C and S2D).

To establish whether the reduction in theta frequency observed in Dp1Tyb mice arose simply from the increased time that those animals spent immobile, we subsequently restricted our analysis to periods of movement only (see STAR Methods). Consistent with the results above, theta

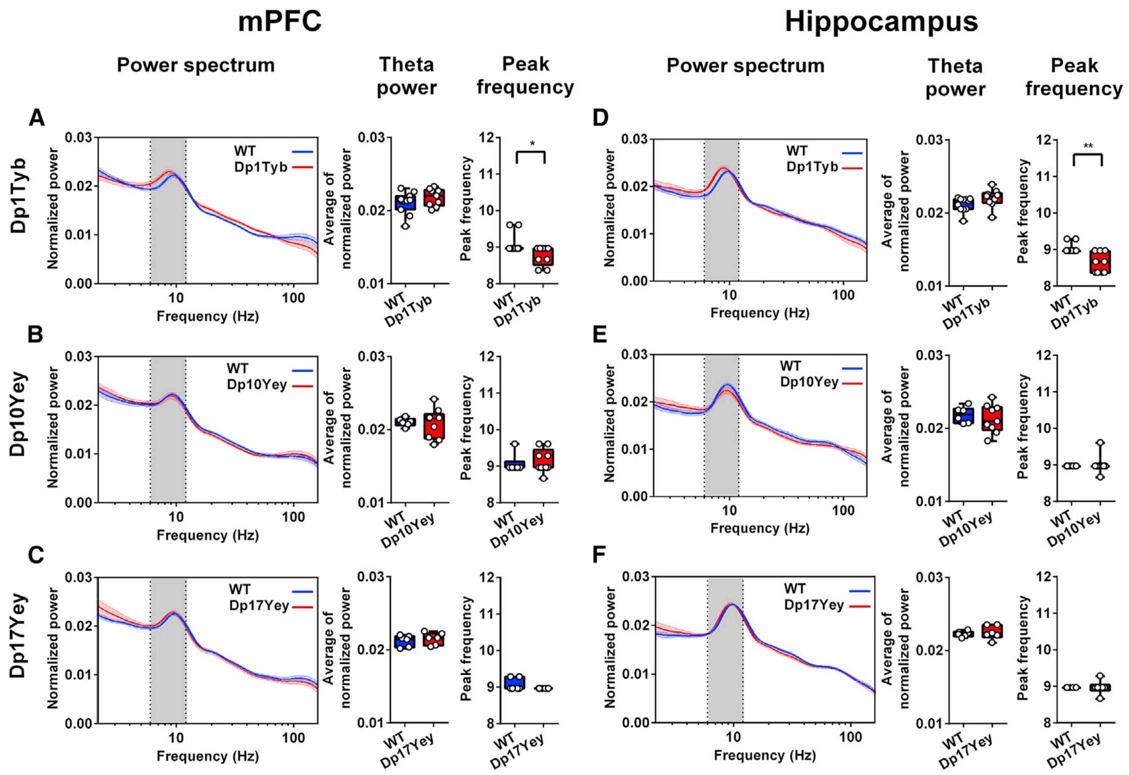

Figure 2. Theta Oscillations in the mPFC and Hippocampus during Spontaneous Alternation

(A–F) Power spectra, mean theta power, and peak theta frequency in the (A–C) mPFC and (D–F) hippocampus for (A and D) Dp1Tyb and WT, (B and E) Dp10Yey and WT, and (C and F) Dp17Yey and WT animals during spontaneous alternation in the T-maze. Grey rectangles indicate the 6–12-Hz theta band. There are no differences in theta power between mutant mice and WT groups in either the mPFC or hippocampus. However, peak theta frequency in Dp1Tyb animals is significantly lower than in WT in both the (A) mPFC (Dp1Tyb: 8.76 ± 0.26 Hz; WT: 9.08 ± 0.26 Hz; Mann-Whitney U = 22.5, $p < 0.05$) and (D) hippocampus (Dp1Tyb: 8.63 ± 0.28 Hz; WT: 9.02 ± 0.13 Hz; Mann-Whitney U = 13.5, $p < 0.005$), but no different in the other mutant mouse groups compared to their control populations (Mann-Whitney U test, all $p > 0.4$).

Data are presented as box-whisker plots indicating the median, 25th and 75th percentiles, and minimum and maximum values, with data for individual mice superimposed.

Please refer to Table S2 for full details of all statistical analyses.

frequency in both the hippocampus and mPFC of Dp1Tyb mice was still significantly lower than that in WT controls when periods of immobility were excluded. Moreover, this resulted from a reduction in the intercept, but not the slope, of the running speed vs. theta frequency relationship in both regions (Figure S3). Intriguingly, no differences in theta power or frequency were observed during the 100-s habituation phase, prior to the start of the task (Figure S6), suggesting that the reduction in theta frequency observed in Dp1Tyb mice was task dependent. However, we did not record tracking data during the habituation phase and so cannot rule out the possibility that differences in movement statistics between cohorts can account for these results. In sum, these data suggest that Dp1Tyb mice, which exhibit slower decision-making, also show a general slowing of theta-band oscillations across hippocampal and medial prefrontal regions during spatial alternation, independent of running speed.

Next, we looked for differences in theta power and frequency between the first and second (sample and choice) runs, which are associated with memory encoding and retrieval, respectively. We identified a significant increase in hippocampal and medial prefrontal theta power during the second run in both the Dp10Yey and WT and the Dp17Yey and WT cohorts (i.e., a main effect, independent of genotype) and a significant interaction between run and genotype on hippocampal theta power in the Dp1Tyb and WT cohorts (Figure S7). Subsequent analysis indicated that this interaction was driven by Dp1Tyb animals showing reduced hippocampal theta power on the second run, while their WT control animals showed increased theta power during the same period, consistent with the other groups. The observed increase in theta power during the choice run is consistent with the involvement of theta oscillations in memory retrieval (Kaplan et al., 2014; Klimesch et al., 2001; Winson, 1978), and it is interesting to note that only Dp1Tyb mice did not show this effect, alongside the reduction in theta frequency described above.

Altered Hippocampal Phase-Amplitude Coupling in Dp1Tyb and Dp10Yey Mice

Coherence between the phase of theta oscillations and the amplitude of concurrent gamma band oscillations is prevalent

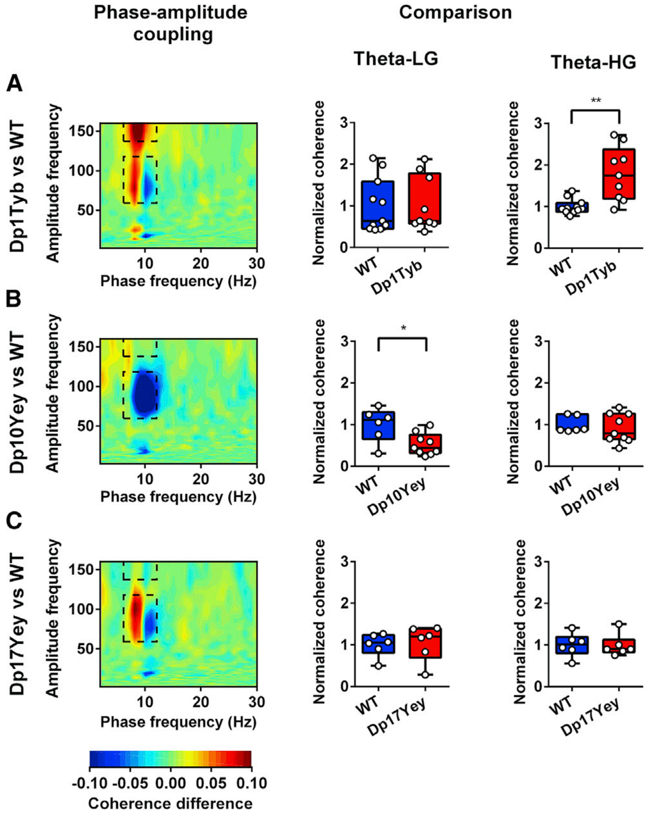

Figure 3. Hippocampal Phase-Amplitude Coupling during Spontaneous Alternation

Left: comodulograms showing differences in hippocampal phase-amplitude coupling between each mutant mouse group and WT, with warm colors indicating stronger coupling in DS mice. These illustrate two prominent peaks: one between the 6–12-Hz theta phase and 60–120-Hz "low gamma" (LG) amplitude and another between the 6–12-Hz theta phase and 140–160-Hz "high gamma" (HG) amplitude (black dashed rectangles; see Figures S8A and S8B for further details). Right: theta-LG and theta-HG cross-frequency coherence values, normalized by the mean value in the corresponding WT control cohort to facilitate comparison.

(A) Dp1Tyb mice show no difference in theta-LG coupling, but significantly greater theta-HG coupling, compared to WT (Mann-Whitney U = 11.0, $p < 0.01$).

(B) Conversely, Dp10Yey mice show significantly lower theta-LG coupling (Mann-Whitney U = 8.0, $p < 0.05$), but no difference in theta-HG coupling, compared to WT.

(C) Finally, Dp17Yey mice show no difference in either theta-LG or theta-HG compared to WT.

Data are presented as box-whisker plots indicating the median, 25th and 75th percentiles, and minimum and maximum values, with data for individual mice superimposed.

Please refer to Table S2 for full details of all statistical analyses.

in the rodent hippocampus (Bragin et al., 1995; Colgin et al., 2009) and across the human neocortex (Canolty et al., 2006). In addition, theta-gamma phase-amplitude coupling (PAC) has been implicated in successful memory function (Lisman, 2005; Tort et al., 2009). Hence, we asked whether the three DS mouse lines exhibited abnormal PAC that might be associated with the observed differences in behavior. Average cross-frequency coherence images across all animals revealed two distinct PAC peaks in the hippocampal LFP: one between 6–12-Hz theta and 60–120-Hz "low gamma" (LG) oscillations and another between 6–12-Hz theta and 140–160-Hz "high gamma" (HG) oscillations (Figure S8A), while theta phase modulation of LG or HG amplitude was entirely absent in the mPFC (Figure S8B).

Interestingly, subsequent analyses indicated that the magnitude of hippocampal PAC in each pair of frequency bands also differed between specific DS models and WT controls. First, we found that theta-HG PAC was significantly increased in the Dp1Tyb group—which exhibited slowed decision-making—relative to WT controls, but not in any other mouse strain (Figure 3A). Second, we found that theta-LG PAC was significantly reduced in the Dp10Yey group—which showed impaired spatial alternation—relative to WT controls, but not in any other strain (Figure 3B). There was no alteration in hippocampal PAC across any pair of frequency bands in Dp17Yey animals, which also exhibit no significant differences in behavior compared to their WT control group (Figure 3C), and no differences in theta-LG or theta-HG PAC between WT cohorts (Figures S2E and S2F). Importantly, the apparent increase in lower theta-band coupling with LG amplitude exhibited by Dp1Tyb and Dp17Yey animals, concomitant with a decrease in higher theta-band coupling with LG amplitude (Figures 3A and 3C), was simply due to differences in peak theta frequency between groups (Figures S8C–S8E). In addition, we found no evidence for a difference in LG or HG power between mutant mice and their WT controls (Figures S9A and S9B).

To confirm that the increased theta-HG PAC observed in Dp1Tyb mice did not arise from differences in movement statistics, we subsequently removed any effect of average time spent immobile on average theta-HG PAC values across animals in both mutant and control groups by linear regression and then compared the residual values between groups. This analysis confirmed that the increased theta-HG PAC in the hippocampus exhibited by Dp1Tyb mice was independent of differences in movement statistics (Figure S3). In sum, these data distinguish changes in hippocampal theta phase modulation of local HG (Dp1Tyb) and LG (Dp10Yey) amplitude in a manner that can be associated with increased trial latency and impaired spatial alternation, respectively.

Increased Hippocampal-mPFC Theta Coherence in Dp1Tyb Mice

Planning, decision-making, memory encoding, and retrieval processes are each associated with increased functional connectivity between the hippocampus and mPFC in both rodents (Benchenane et al., 2010, 2011; Jones and Wilson, 2005; Siapas et al., 2005; Young and McNaughton, 2009) and humans (Guitart-Masip et al., 2013; Kaplan et al., 2014). Interestingly, abnormalities in functional connectivity have also been implicated in various neurodevelopmental disorders, including DS (Anderson et al., 2013; Vega et al., 2015). Hence, we next examined theta and gamma band coherence between the hippocampus and mPFC, with the hypothesis that differences in functional connectivity between those regions might be associated with the cognitive impairments observed in these DS mice.

First, we found that theta coherence between the hippocampus and mPFC was significantly greater in Dp1Tyb mice

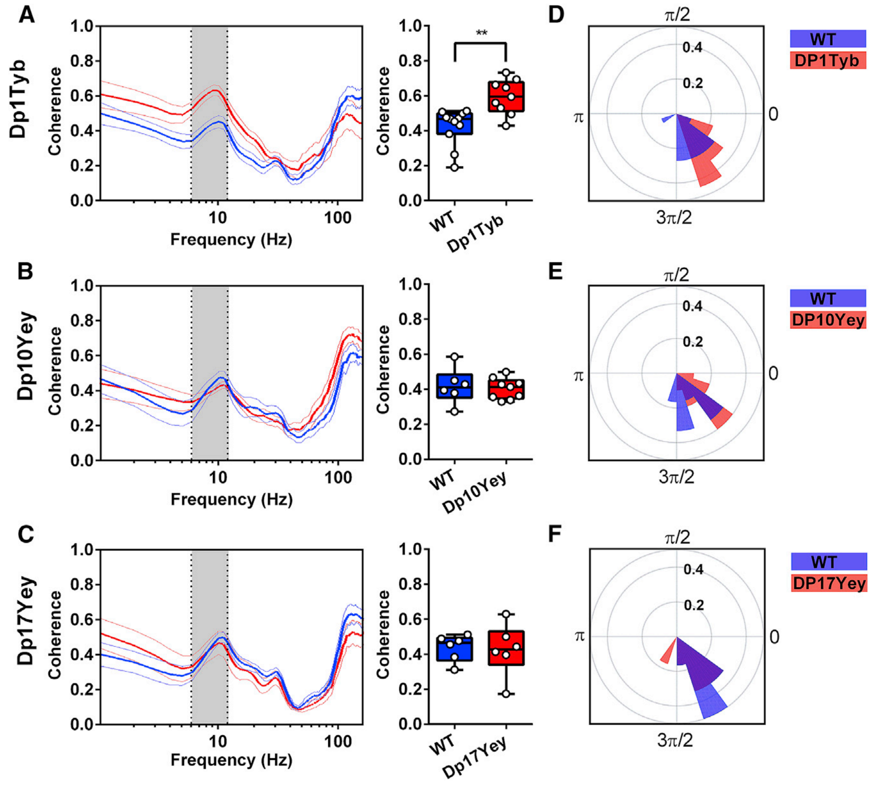

Figure 4. Hippocampal-Medial Prefrontal Phase Coupling during Spontaneous Alternation

(A–C) Coherence spectra and mean theta-band coherence illustrating hippocampal-mPFC phase coupling during spontaneous alternation behavior. Grey rectangles indicate the 6–12-Hz theta band.

(A) Theta-band coherence is significantly greater in Dp1Tyb mice compared to WT (Mann-Whitney U = 11.0, $p < 0.005$), while there is no difference between either (B) Dp10Yey and WT or (C) Dp17Yey and WT animals.

(D–F) Circular mean phase offset between the mPFC and hippocampus for (D) Dp1Tyb and WT, (E) Dp10Yey and WT, and (F) Dp17yey and WT animals. The radial axis shows relative frequency, and the polar axis indicates the circular mean theta phase difference between the mPFC and hippocampus. These results suggest that hippocampal theta oscillations lead those in the mPFC by ~1 radian (equivalent to ~20 ms at 9 Hz) in all mutant and WT mice, without any differences between groups (Watson-Williams test, all $p > 0.07$).

Data are presented as box-whisker plots indicating the median, 25th and 75th percentiles, and minimum and maximum values, with data for individual mice superimposed.

Please refer to Table S2 for full details of all statistical analyses.

compared to WT littermates (Figure 4A), while no such differences were observed between Dp10Yey or Dp17Yey mice and their control groups (Figures 4B and 4C) or between WT cohorts (Figure S2G). In addition, there were no differences in either LG or HG coherence between the hippocampus and mPFC in any mutant mouse group compared to their WT controls (Figure S9C). To confirm that the increased theta coherence observed in Dp1Tyb mice, compared to their WT littermates, did not simply arise due to the observed differences in movement statistics, we again removed any effect of average time spent immobile on theta coherence values across animals in both groups by linear regression and then compared the residual values between groups (Figure S3). This confirmed that the increased theta coherence exhibited by Dp1Tyb mice was independent of differences in movement statistics.

To further characterize potential changes in functional connectivity across mouse lines, we extracted the theta phase lag between the hippocampus and mPFC in order to assess the direction of communication between these regions (Figures 4D–4F). In each group of animals, we found that hippocampal theta oscillations led those in the mPFC by $\sim$1 radian, which is equivalent to $\sim$20 ms for a 6–12-Hz theta oscillation, without any difference between strains. Intriguingly, these results indicate that Dp1Tyb mice—which exhibit slowed planning and decision-making behavior during the spatial alternation task—showed increased theta-band coherence between the hippocampus and mPFC, without any differences in the direction of communication between those regions. This indicates that the cognitive dysfunction observed in Dp1Tyb animals is associated with an increased influence of hippocampal inputs on medial prefrontal dynamics, rather than with changes in the direction of information flow between those regions per se.

Behavioral and LFP Characteristics Are Preserved across the Lifespan in DS Mouse Models

Finally, we asked whether the behavioral and LFP abnormalities observed in Dp1Tyb and Dp10Yey mice persisted throughout life or were specific to the adolescent period during which they were initially tested (Foster et al., 2007). To this end, we repeated tests of alternation behavior and recorded LFP data from the same animals at 6 and 9 months of age, alongside age-matched WT controls (see Figure S1C for further details; Table S3 for animal and trial numbers). Importantly, we found that the differences in both behavior and neural dynamics described above remained stable throughout this long-term assessment period.

First, we found that trial latency was significantly greater in Dp1Tyb mice compared to their WT control group across all three time points (Figure 5A), and the observed reduction in both hippocampal and mPFC peak theta frequency also persisted with age (Figures 5B and 5C). Similarly, hippocampal theta-HG PAC was significantly greater in Dp1Tyb mice compared to WT at all ages (Figure 5D), and theta coherence between the hippocampus and mPFC remained significantly higher than in the WT across the lifespan (Figure 5E).

Second, we found that the impaired alternation rate observed in young Dp10Yey mice persisted with age (Figure 5F). In contrast to WT mice, the alternation rate in Dp10Yey mice was not different from chance at any time point. Similarly,

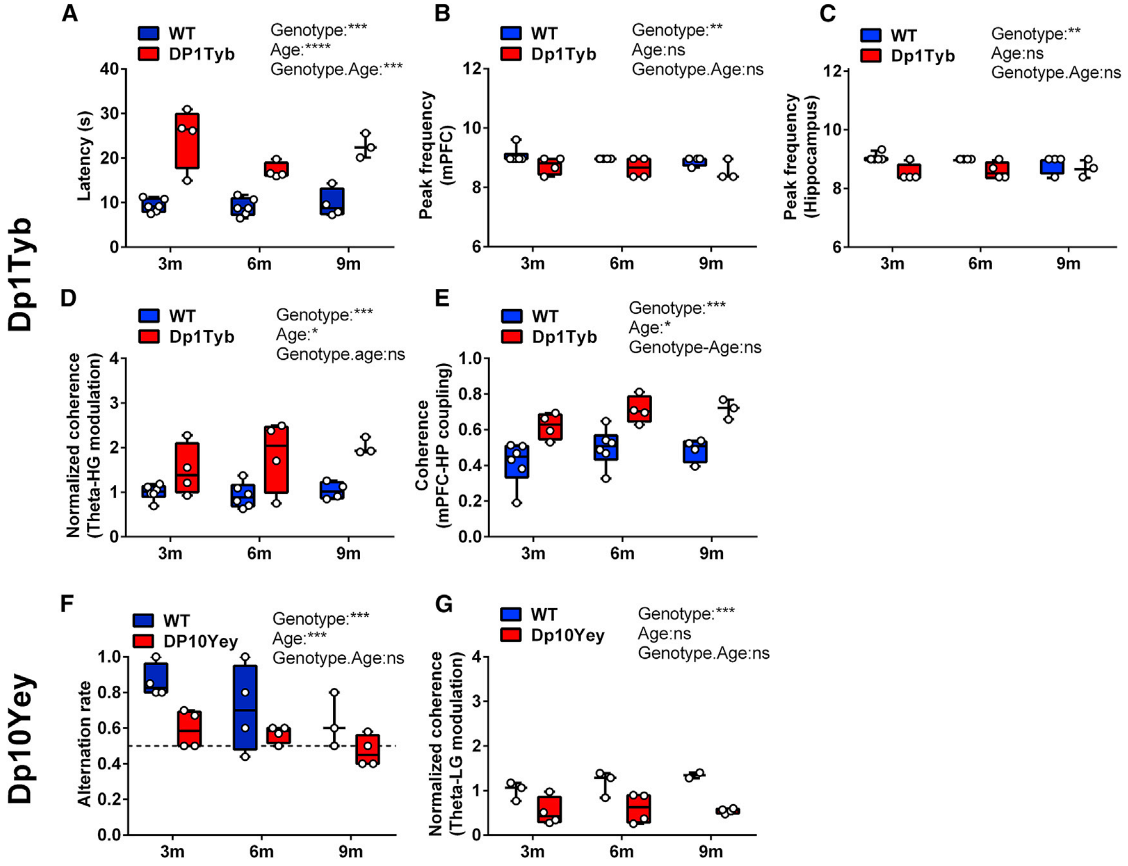

Figure 5. Behavioral and Electrophysiological Data across the Lifespan

Behavioral and LFP data at 3–4 months (3 m), 6–7 months (6 m), and 9–10 months (9 m) of age in (A–E) Dp1Tyb and (F and G) Dp10Yey mice.

(A) Trial latency remains significantly greater in Dp1Tyb mice compared to WT throughout the lifespan (generalized linear model [GLM]; type III tests $\chi^2(1) = 56.1$, $p < 0.0001$).

(B and C) Similarly, peak theta frequency in both the (B) mPFC (GLM; type III $\chi^2(1) = 6.84$, $p < 0.01$) and (C) hippocampus (GLM; type III $\chi^2(1) = 8.93$, $p < 0.01$) is shifted to a significantly lower frequency.

(D and E) Hippocampal theta-HG phase-amplitude coupling (GLM; type III $\chi^2(1) = 14.2$, $p < 0.0001$) (D) and theta coherence between the mPFC and hippocampus (GLM; type III $\chi^2(1) = 29.6$, $p < 0.0001$) (E) are also increased in Dp1Tyb mice at all three time points, compared to their WT control group.

(F) Alternation rate remains significantly lower in Dp10Yey mice compared to WT throughout the lifespan (GLM; type III $\chi^2(1) = 12.5$, $p < 0.0001$) and does not differ from chance level (black dashed line) at any age (Friedman's test, $\chi^2(5) = 8.2$, $p > 0.15$), while the WT control group consistently performs above chance (Friedman's $\chi^2(5) = 10.3$, $p < 0.05$).

(G) Hippocampal theta-LG phase-amplitude coupling is also significantly lower in Dp10Tyb mice at all three time points (GLM; type III $\chi^2(1) = 18.2$, $p < 0.0001$). Data are presented as box-whisker plots indicating the median, 25th and 75th percentiles, and minimum and maximum values, with data for individual mice superimposed.

Please refer to Table S2 for full details of all statistical analyses.

hippocampal theta-LG PAC remained consistently lower in Dp10Yey mice relative to WT controls (Figure 5G). In sum, these results suggest that the observed differences in behavior and neural dynamics between these DS mouse models and their WT control groups generally remained stable throughout adulthood, suggesting that aging neither alleviated nor worsened the phenotype in either strain.

DISCUSSION

The present study reveals distinct cognitive deficits and electrophysiological differences in three mouse models of DS, which, combined, carry duplications covering all Hsa21-orthologous regions. By taking an unbiased approach, we aimed to discover if cognitive deficits resulting from the triplication of genes/DNA elements in Hsa21 could be linked to individual regions with different sequence contents. As a measure of cognitive function, we used a canonical test of spatial working memory: spontaneous alternation in a T-maze (Lalonde, 2002). This behavioral test probes both decision-making and mnemonic function, based on the premise that mice have evolved an optimal strategy to explore their environment that relies on memorizing previous trajectories and then using that information to plan future trajectories. Numerous cortical regions are implicated in the successful performance of this task, most notably the hippocampus and mPFC (Deacon

and Rawlins, 2006; Lalonde, 2002; Sarnyai et al., 2000; Wenk, 2001).

Using this behavioral paradigm, we have shown that alternation deficits and hippocampal/mPFC neural dysfunction segregate with different regions of homology in the DS models. First, we found that spatial alternation, a putative index of mnemonic function, was impaired in Dp10Yey mice. In contrast, trial latency, which provides an independent measure of cognitive processing that includes decision-making, planning, goal-directed behavior, and attention (Bizon et al., 2012; Pioli et al., 2014), was prolonged in Dp1Tyb mice. In addition, we have shown that Dp1Tyb mice have a lower peak frequency in the theta band in the hippocampus and mPFC, an increase in PAC between theta and HG in the hippocampus, and a striking increase in theta phase coupling between the mPFC and hippocampus—each of which is independent of the observed differences in movement statistics between those animals and their WT littermates. Conversely, Dp10Yey mice exhibit decreased PAC between theta and LG in the hippocampus, while Dp17Yey mice did not show any significant behavioral deficits in spatial alternation or any alteration in the electrophysiology of the hippocampus or mPFC. Crucially, the alterations in behavior and neural dynamics observed in our mutant mice are also unlikely to arise from differences in intrauterine environment, rearing, or housing conditions, as we found no differences between WT littermate groups either behaviorally or physiologically.

Previous studies that have interrogated hippocampal function in similar mutant mouse populations (Aziz et al., 2018; Levenga et al., 2018) have found no impairments of mnemonic function in Dp10Yey mice (Belichenko et al., 2015; Yu et al., 2010b). In contrast, we observed decreased alternation rates in Dp10Yey mice suggestive of a spatial working memory deficit (Deacon and Rawlins, 2006). These conflicting findings likely reflect subtle differences in the behavioral tasks employed, which emphasize complementary aspects of neural processing both within the hippocampus and among a wider network of functionally integrated brain regions, and should be the subject of further investigation (D'Hooge and De Deyn, 2001; Reisel et al., 2002; Sanderson and Bannerman, 2012). The behavioral phenotype observed here was associated with a decrease in theta-gamma PAC in the hippocampus. It has been well established that working memory relies on the periodic reactivation of encoded information by theta modulation of gamma oscillations in rodents (Benchenane et al., 2011; Tort et al., 2008, 2010) and humans (Jensen and Lisman, 2005; Leszczyński et al., 2015; Lisman and Idiart, 1995; Maris et al., 2011; Poch et al., 2011), and so our finding of decreased gamma-theta coupling in Dp10Yey mice is consistent with their behavioral phenotype and indicates specific abnormalities of hippocampal circuitry in this model. Our data may thus provide a functional basis for the memory problems evident in people with DS (Grieco et al., 2015; Lanfranchi et al., 2010; Rowe et al., 2006). Dp10Yey mice were generated to carry an internal duplication spanning the 39 Hsa21 protein-coding orthologs mapping to Mmu10, and several of these genes—such as *ADAR2*, *S100B*, *CSTB*, *PRMT2*, and *TRPM2*—have been shown to play a role in brain development and function, such that aberrant gene dosage may be related to intellectual disability in DS (Block et al., 2015; Gupta et al., 2016).

An unexpected finding of this study was the delayed decision-making observed in Dp1Tyb mice with preserved memory function. Similar behavioral differences have also been found in humans with DS, who exhibit markedly slower reaction times (Brunamonti et al., 2011; Inui et al., 1995; Vicari et al., 2000). This impairment has been attributed to deficits in executive function that involve information processing, attention, and inhibition (Grieco et al., 2015; Rowe et al., 2006), resulting in difficulty prioritizing, staying engaged with a task, and consistently responding in the same manner to certain situations (Costanzo et al., 2013; Rowe et al., 2006). Importantly, we found that the increased trial latency observed in these animals was associated with a reduction in theta frequency across both the hippocampus and mPFC. It is well established that theta frequency is correlated with running speed in rodents (Jeewajee et al., 2008), and so a potential explanation for both of these findings is that Dp1Tyb mice simply moved more slowly in general. However, although we found that Dp1Tyb mice spent more time immobile—presumably, reflecting their inability to retain focus on the task—they exhibited no differences in running speed compared to their WT littermates, and the observed reduction in theta frequency was still present when we restricted our analyses to movement periods only.

In addition, we found that the delayed decision-making in Dp1Tyb mice was associated with increased hippocampal-mPFC theta coherence. Communication between the mPFC and hippocampus occurs through both direct projections and bidirectional pathways via intermediaries in the thalamus, perirhinal, and lateral entorhinal cortices (Thierry et al., 2000; Varela et al., 2014; Zhong et al., 2006). It is well accepted that coherence of neuronal activity across brain regions serves as a general mechanism for increasing effective communication during memory and attention tasks. Hippocampal-prefrontal theta-band synchrony facilitates hippocampal inputs to the mPFC and the integration of gamma-mediated cell assemblies in the mPFC (Colgin, 2011; Sirota et al., 2008). In addition, theta-band synchrony has frequently been observed during the "deliberative" phase of T-maze tasks in rodents (Benchenane et al., 2010; Jones and Wilson, 2005; Lalonde, 2002), as well as during more conventional decision-making tasks in humans (Guitart-Masip et al., 2013). Our results therefore suggest that Dp1Tyb mice engage in more prolonged or pronounced deliberation prior to choosing an arm of the T-maze, although this does not necessarily lead to a poorer outcome, as those animals tended to make the correct choice (i.e., exhibit spatial alternation) once a decision had been made. Hence, our finding of increased hippocampal-mPFC theta coherence is consistent with the observed behavioral phenotype. Widespread increases in low-frequency coherence between distributed brain networks, particularly including the mPFC, are also observed in people with DS, are more evident in DS patients than in patients with other neurological disorders, and are inversely related to cognitive performance (Anderson et al., 2013).

Alternatively, it is possible that both the increased time spent immobile and the increased hippocampal-mPFC theta coherence exhibited by Dp1Tyb animals could be accounted for by

OPEN ACCESS
CellPress

an increase in generalized anxiety (Adhikari et al., 2010). This interpretation is unlikely, however, as a parallel study has found no evidence for differences in anxiety between Dp1Tyb and WT mice on the elevated plus maze (M. G, unpublished data). Moreover, previous research has demonstrated that anxiogenic environments such as the elevated plus maze produce increased theta coherence between the mPFC and ventral, rather than dorsal, hippocampus, in contrast to the results presented here.

Dyrk1A, located on chromosome 21, is a major candidate protein-coding gene for several aspects of DS and encodes a kinase involved in neurodevelopment (Arron et al., 2006; Fotaki et al., 2002; Guimera et al., 1999; Hämmerle et al., 2008; Park et al., 2009; Tejedor and Hämmerle, 2011). Overexpression of this gene in transgenic mice results in changes in inhibitory circuits in the mPFC (Ruiz-Mejias et al., 2016; Souchet et al., 2014) and may result in abnormal neural dynamics, particularly in the gamma band. Furthermore, *Dyrk1a* overexpression in mice induces learning and memory impairments detectable in the Morris water maze and Y-maze (Souchet et al., 2014). Here, we showed that reducing *Dyrk1a* to the normal two copies in Dp1Tyb mice failed to rescue the prolonged decision-making we observed in the spatial alternation task. Thus, *Dyrk1a* overexpression is not required for this phenotype, leading us to conclude that another gene or genes, when present in three copies within the Dp1Tyb region, are involved in the abnormal decision-making behavior described here. This is an important result that may, in part, explain why most of the current competitive *Dyrk1a* inhibitors fail to pass the pre-clinical stage with respect to improvement of cognitive impairments in DS (Neumann et al., 2018). Of the 148 protein-coding genes within the region duplicated in Dp1Tyb mice, a handful are candidates for further exploration.

We note that there may also be critical effects from dosage sensitivity of non-protein coding elements on Hsa21, and our genetically unbiased approach will allow us to map to the DNA region, not just to focus on the relatively limited set of protein-coding elements for which we have functional information. Finding the genes (coding and non-coding) responsible for the cognitive and electrophysiological phenotypes observed in these mice has the translational potential to reveal important routes toward phenotype modifying therapies, for example, by antisense oligomers, but our results indicate that targeting a single gene is unlikely to be sufficient.

In summary, our study elucidates an important link among different regions of Hsa21, cognitive deficits, and both local and long-range neural circuit dysfunction. Importantly, our results imply that specific cognitive deficits in DS may result from different underlying genetic, functional, and regional abnormalities. This has important implications for understanding such cognitive deficits and indicates that therapies in DS will likely need to target multiple processes.

STAR★METHODS

Detailed methods are provided in the online version of this paper and include the following:

- KEY RESOURCES TABLE
- LEAD CONTACT AND MATERIALS AVAILABILITY
- EXPERIMENTAL MODEL AND SUBJECT DETAILS
- METHOD DETAILS
  - Surgical Preparation and Transmitter Implantation for Long-term Recording
  - Behavioral Testing: T-maze Spontaneous Alternation
  - Histology
  - EEG Data Analysis
- QUANTIFICATION AND STATISTICAL ANALYSIS
- DATA AND CODE AVAILABILITY

SUPPLEMENTAL INFORMATION

Supplemental Information can be found online at https://doi.org/10.1016/j.celrep.2019.12.065.

ACKNOWLEDGMENTS

E.M.C.F. and V.L.J.T. were supported by grants from the Wellcome Trust (grants 080174, 098327, and 098328). V.L.J.T. was also supported by MRC programme U117527252 and by the Francis Crick Institute, which receives its core funding from the UK Medical Research Council (FC001194), Cancer Research UK, (FC001194), and the Wellcome Trust (FC001194). N.B. is supported by a Wellcome Trust Principal Research Fellowship (202805/Z/16/Z). We thank Eugene Yu and Mariona Arbones for mouse strains, Nick Rawlins for comments on an earlier version of the manuscript, and Andre Strydom for constructive input. We also thank Dr Karen Cleverly and Ms. Dorota Gibbins for colony management and genotyping and the Biological Research Facility of the Francis Crick Institute for animal husbandry.

AUTHOR CONTRIBUTIONS

P.C. collected behavioral and electrophysiology data; P.C., D.B., and N.S. analyzed electrophysiology data; P.C., N.S., and S.N. analyzed histology data; F.K.W., V.L.J.T., and E.M.C.F. provided mice for this study; S.S., M.G., T.C., N.B., M.C.W., and E.M.C.F. helped direct the study; and all authors contributed to the manuscript, which was primarily drafted by P.C., D.B., M.C.W., and E.M.C.F.

DECLARATION OF INTERESTS

The authors declare no competing interests.

Received: May 7, 2019
Revised: October 2, 2019
Accepted: December 17, 2019
Published: January 28, 2020

REFERENCES

Adhikari, A., Topiwala, M.A., and Gordon, J.A. (2010). Synchronized activity between the ventral hippocampus and the medial prefrontal cortex during anxiety. Neuron *65*, 257–269.

Anderson, J.S., Nielsen, J.A., Ferguson, M.A., Burback, M.C., Cox, E.T., Dai, L., Gerig, G., Edgin, J.O., and Korenberg, J.R. (2013). Abnormal brain synchrony in Down Syndrome. Neuroimage Clin. *2*, 703–715.

Arron, J.R., Winslow, M.M., Polleri, A., Chang, C.P., Wu, H., Gao, X., Neilson, J.R., Chen, L., Heit, J.J., Kim, S.K., et al. (2006). NFAT dysregulation by increased dosage of DSCR1 and DYRK1A on chromosome 21. Nature *441*, 595–600.

Aziz, N.M., Guedj, F., Pennings, J.L.A., Olmos-Serrano, J.L., Siegel, A., Haydar, T.F., and Bianchi, D.W. (2018). Lifespan analysis of brain development, gene expression and behavioral phenotypes in the Ts1Cje, Ts65Dn and

Dp(16)1/Yey mouse models of Down syndrome. Dis. Model. Mech. *11*, dmm031013.

Belichenko, P.V., Kleschevnikov, A.M., Becker, A., Wagner, G.E., Lysenko, L.V., Yu, Y.E., and Mobley, W.C. (2015). Down Syndrome Cognitive Phenotypes Modeled in Mice Trisomic for All HSA 21 Homologues. PLoS ONE *10*, e0134861.

Benchenane, K., Peyrache, A., Khamassi, M., Tierney, P.L., Gioanni, Y., Battaglia, F.P., and Wiener, S.I. (2010). Coherent theta oscillations and reorganization of spike timing in the hippocampal- prefrontal network upon learning. Neuron *66*, 921–936.

Benchenane, K., Tiesinga, P.H., and Battaglia, F.P. (2011). Oscillations in the prefrontal cortex: a gateway to memory and attention. Curr. Opin. Neurobiol. *21*, 475–485.

Berens, P. (2009). CircStat: A MATLAB toolbox for circular statistics. J. Stat. Softw. *1*, 2009.

Bizon, J.L., Foster, T.C., Alexander, G.E., and Glisky, E.L. (2012). Characterizing cognitive aging of working memory and executive function in animal models. Front. Aging Neurosci. *4*, 19.

Block, A., Ahmed, M.M., Dhanasekaran, A.R., Tong, S., and Gardiner, K.J. (2015). Sex differences in protein expression in the mouse brain and their perturbations in a model of Down syndrome. Biol. Sex Differ. *6*, 24.

Bohbot, V.D., Copara, M.S., Gotman, J., and Ekstrom, A.D. (2017). Low-frequency theta oscillations in the human hippocampus during real-world and virtual navigation. Nat. Commun. *8*, 14415.

Bragin, A., Jandó, G., Nádasdy, Z., Hetke, J., Wise, K., and Buzsáki, G. (1995). Gamma (40-100 Hz) oscillation in the hippocampus of the behaving rat. J. Neurosci. *15*, 47–60.

Brunamonti, E., Pani, P., Papazachariadis, O., Onorati, P., Albertini, G., and Ferraina, S. (2011). Cognitive control of movement in Down syndrome. Res. Dev. Disabil. *32*, 1792–1797.

Buzsáki, G., and Chrobak, J.J. (1995). Temporal structure in spatially organized neuronal ensembles: a role for interneuronal networks. Curr. Opin. Neurobiol. *5*, 504–510.

Canolty, R.T., Edwards, E., Dalal, S.S., Soltani, M., Nagarajan, S.S., Kirsch, H.E., Berger, M.S., Barbaro, N.M., and Knight, R.T. (2006). High gamma power is phase-locked to theta oscillations in human neocortex. Science *313*, 1626–1628.

Chang, P., Hashemi, K.S., and Walker, M.C. (2011). A novel telemetry system for recording EEG in small animals. J. Neurosci. Methods *201*, 106–115.

Chang, P., Fabrizi, L., Olhede, S., and Fitzgerald, M. (2016). The development of nociceptive network activity in the somatosensory cortex of freely moving rat pups. Cereb. Cortex *26*, 4513–4523.

Clark, C.A.C., Fernandez, F., Sakhon, S., Spanò, G., and Edgin, J.O. (2017). The medial temporal memory system in Down syndrome: Translating animal models of hippocampal compromise. Hippocampus *27*, 683–691.

Colgin, L.L. (2011). Oscillations and hippocampal-prefrontal synchrony. Curr. Opin. Neurobiol. *21*, 467–474.

Colgin, L.L., Denninger, T., Fyhn, M., Hafting, T., Bonnevie, T., Jensen, O., Moser, M.-B., and Moser, E.I. (2009). Frequency of gamma oscillations routes flow of information in the hippocampus. Nature *462*, 353–357.

Costanzo, F., Varuzza, C., Menghini, D., Addona, F., Gianesini, T., and Vicari, S. (2013). Executive functions in intellectual disabilities: a comparison between Williams syndrome and Down syndrome. Res. Dev. Disabil. *34*, 1770–1780.

D'Hooge, R., and De Deyn, P.P. (2001). Applications of the Morris water maze in the study of learning and memory. Brain Res. Brain Res. Rev. *36*, 60–90.

de Chaumont, F., Dallongeville, S., Chenouard, N., Hervé, N., Pop, S., Provoost, T., Meas-Yedid, V., Pankajakshan, P., Lecomte, T., Le Montagner, Y., et al. (2012). Icy: an open bioimage informatics platform for extended reproducible research. Nat. Methods *9*, 690–696.

de Graaf, G., Buckley, F., and Skotko, B.G. (2015). Estimates of the live births, natural losses, and elective terminations with Down syndrome in the United States. Am. J. Med. Genet. A. *167A*, 756–767.

Deacon, R.M.J., and Rawlins, J.N.P. (2006). T-maze alternation in the rodent. Nat. Protoc. *1*, 7–12.

Duchon, A., and Herault, Y. (2016). DYRK1A, a dosage-sensitive gene involved in neurodevelopmental disorders, is a target for drug development in Down syndrome. Front. Behav. Neurosci. *10*, 104.

Fell, J., and Axmacher, N. (2011). The role of phase synchronization in memory processes. Nat. Rev. Neurosci. *12*, 105–118.

Foster, H.L., Small, J.D., and Fox, J.G. (2007). The mouse in biomedical research (Elsevier).

Fotaki, V., Dierssen, M., Alcántara, S., Martínez, S., Martí, E., Casas, C., Visa, J., Soriano, E., Estivill, X., and Arbonés, M.L. (2002). Dyrk1A haploinsufficiency affects viability and causes developmental delay and abnormal brain morphology in mice. Mol. Cell. Biol. *22*, 6636–6647.

Grieco, J., Pulsifer, M., Seligsohn, K., Skotko, B., and Schwartz, A. (2015). Down syndrome: Cognitive and behavioral functioning across the lifespan. Am. J. Med. Genet. C. Semin. Med. Genet. *169*, 135–149.

Griesmayr, B., Gruber, W.R., Klimesch, W., and Sauseng, P. (2010). Human frontal midline theta and its synchronization to gamma during a verbal delayed match to sample task. Neurobiol. Learn. Mem. *93*, 208–215.

Guderian, S., Schott, B.H., Richardson-Klavehn, A., and Düzel, E. (2009). Medial temporal theta state before an event predicts episodic encoding success in humans. Proc. Natl. Acad. Sci. USA *106*, 5365–5370.

Guimera, J., Casas, C., Estivill, X., and Pritchard, M. (1999). Human minibrain homologue (MNBH/DYRK1): characterization, alternative splicing, differential tissue expression, and overexpression in Down syndrome. Genomics *57*, 407–418.

Guitart-Masip, M., Barnes, G.R., Horner, A., Bauer, M., Dolan, R.J., and Duzel, E. (2013). Synchronization of medial temporal lobe and prefrontal rhythms in human decision making. J. Neurosci. *33*, 442–451.

Gupta, M., Dhanasekaran, A.R., and Gardiner, K.J. (2016). Mouse models of Down syndrome: gene content and consequences. Mamm. Genome *27*, 538–555.

Hämmerle, B., Elizalde, C., and Tejedor, F.J. (2008). The spatio-temporal and subcellular expression of the candidate Down syndrome gene Mnb/Dyrk1A in the developing mouse brain suggests distinct sequential roles in neuronal development. Eur. J. Neurosci. *27*, 1061–1074.

Hanney, M., Prasher, V., Williams, N., Jones, E.L., Aarsland, D., Corbett, A., Lawrence, D., Yu, L.M., Tyrer, S., Francis, P.T., et al.; MEADOWS trial researchers (2012). Memantine for dementia in adults older than 40 years with Down's syndrome (MEADOWS): a randomised, double-blind, placebo-controlled trial. Lancet *379*, 528–536.

Harrison, F.E., Hosseini, A.H., and McDonald, M.P. (2009). Endogenous anxiety and stress responses in water maze and Barnes maze spatial memory tasks. Behav. Brain Res. *198*, 247–251.

Hasselmo, M.E., Bodelón, C., and Wyble, B.P. (2002). A proposed function for hippocampal theta rhythm: separate phases of encoding and retrieval enhance reversal of prior learning. Neural Comput. *14*, 793–817.

Inui, N., Yamanishi, M., and Tada, S. (1995). Simple reaction times and timing of serial reactions of adolescents with mental retardation, autism, and Down syndrome. Percept. Mot. Skills *81*, 739–745.

Jeewajee, A., Barry, C., O'Keefe, J., and Burgess, N. (2008). Grid cells and theta as oscillatory interference: electrophysiological data from freely moving rats. Hippocampus *18*, 1175–1185.

Jensen, O., and Lisman, J.E. (2005). Hippocampal sequence-encoding driven by a cortical multi-item working memory buffer. Trends Neurosci. *28*, 67–72.

Jones, M.W., and Wilson, M.A. (2005). Theta rhythms coordinate hippocampal-prefrontal interactions in a spatial memory task. PLoS Biol. *3*, e402.

Kaplan, R., Bush, D., Bonnefond, M., Bandettini, P.A., Barnes, G.R., Doeller, C.F., and Burgess, N. (2014). Medial prefrontal theta phase coupling during spatial memory retrieval. Hippocampus *24*, 656–665.

Klimesch, W., Doppelmayr, M., Yonelinas, A., Kroll, N.E.A., Lazzara, M., Röhm, D., and Gruber, W. (2001). Theta synchronization during episodic retrieval:

neural correlates of conscious awareness. Brain Res. Cogn. Brain Res. *12*, 33–38.

Lalonde, R. (2002). The neurobiological basis of spontaneous alternation. Neurosci. Biobehav. Rev. *26*, 91–104.

Lana-Elola, E., Watson-Scales, S., Slender, A., Gibbins, D., Martineau, A., Douglas, C., Mohun, T., Fisher, E.M., and Tybulewicz, V.Lj. (2016). Genetic dissection of Down syndrome-associated congenital heart defects using a new mouse mapping panel. eLife *5*, e11614.

Lanfranchi, S., Jerman, O., Dal Pont, E., Alberti, A., and Vianello, R. (2010). Executive function in adolescents with Down Syndrome. J. Intellect. Disabil. Res. *54*, 308–319.

Lavenex, P.B., Bostelmann, M., Brandner, C., Costanzo, F., Fragnière, E., Klencklen, G., Lavenex, P., Menghini, D., and Vicari, S. (2015). Allocentric spatial learning and memory deficits in Down syndrome. Front. Psychol. *6*, 62.

Leszczyński, M., Fell, J., and Axmacher, N. (2015). Rhythmic working memory activation in the human hippocampus. Cell Rep. *13*, 1272–1282.

Levenga, J., Peterson, D.J., Cain, P., and Hoeffer, C.A. (2018). Sleep behavior and EEG oscillations in aged Dp(16)1Yey/+ mice: A Down syndrome model. Neuroscience *376*, 117–126.

Lisman, J. (2005). The theta/gamma discrete phase code occuring during the hippocampal phase precession may be a more general brain coding scheme. Hippocampus *15*, 913–922.

Lisman, J.E., and Idiart, M.A. (1995). Storage of 7 +/- 2 short-term memories in oscillatory subcycles. Science *267*, 1512–1515.

Loane, M., Morris, J.K., Addor, M.C., Arriola, L., Budd, J., Doray, B., Garne, E., Gatt, M., Haeusler, M., Khoshnood, B., et al. (2013). Twenty-year trends in the prevalence of Down syndrome and other trisomies in Europe: impact of maternal age and prenatal screening. Eur. J. Hum. Genet. *21*, 27–33.

Lott, I.T., and Dierssen, M. (2010). Cognitive deficits and associated neurological complications in individuals with Down's syndrome. Lancet Neurol. *9*, 623–633.

Maris, E., van Vugt, M., and Kahana, M. (2011). Spatially distributed patterns of oscillatory coupling between high-frequency amplitudes and low-frequency phases in human iEEG. Neuroimage *54*, 836–850.

Nadel, L. (2003). Down's syndrome: a genetic disorder in biobehavioral perspective. Genes Brain Behav. *2*, 156–166.

Nelson, L., Johnson, J.K., Freedman, M., Lott, I., Groot, J., Chang, M., Milgram, N.W., and Head, E. (2005). Learning and memory as a function of age in Down syndrome: a study using animal-based tasks. Prog. Neuropsychopharmacol. Biol. Psychiatry *29*, 443–453.

Neumann, F., Gourdain, S., Albac, C., Dekker, A.D., Bui, L.C., Dairou, J., Schmitz-Afonso, I., Hue, N., Rodrigues-Lima, F., Delabar, J.M., et al. (2018). DYRK1A inhibition and cognitive rescue in a Down syndrome mouse model are induced by new fluoro-DANDY derivatives. Sci. Rep. *8*, 2859.

O'Keefe, J., and Nadel, L. (1978). The Hippocampus as a cognitive map (Clarendon Press).

Park, J., Song, W.J., and Chung, K.C. (2009). Function and regulation of Dyrk1A: towards understanding Down syndrome. Cell. Mol. Life Sci. *66*, 3235–3240.

Paxinos, G.F.K. (2012). The mouse brain in stereotaxic coordinates (Academic Press).

Pennington, B.F., Moon, J., Edgin, J., Stedron, J., and Nadel, L. (2003). The neuropsychology of Down syndrome: evidence for hippocampal dysfunction. Child Dev. *74*, 75–93.

Pioli, E.Y., Gaskill, B.N., Gilmour, G., Tricklebank, M.D., Dix, S.L., Bannerman, D., and Garner, J.P. (2014). An automated maze task for assessing hippocampus-sensitive memory in mice. Behav. Brain Res. *261*, 249–257.

Poch, C., Fuentemilla, L., Barnes, G.R., and Düzel, E. (2011). Hippocampal theta-phase modulation of replay correlates with configural-relational short-term memory performance. J. Neurosci. *31*, 7038–7042.

Raghavachari, S., Kahana, M.J., Rizzuto, D.S., Caplan, J.B., Kirschen, M.P., Bourgeois, B., Madsen, J.R., and Lisman, J.E. (2001). Gating of human theta oscillations by a working memory task. J. Neurosci. *21*, 3175–3183.

Reisel, D., Bannerman, D.M., Schmitt, W.B., Deacon, R.M.J., Flint, J., Borchardt, T., Seeburg, P.H., and Rawlins, J.N.P. (2002). Spatial memory dissociations in mice lacking GluR1. Nat. Neurosci. *5*, 868–873.

Richard, G.R., Titiz, A., Tyler, A., Holmes, G.L., Scott, R.C., and Lenck-Santini, P.P. (2013). Speed modulation of hippocampal theta frequency correlates with spatial memory performance. Hippocampus *23*, 1269–1279.

Rowe, J., Lavender, A., and Turk, V. (2006). Cognitive executive function in Down's syndrome. Br. J. Clin. Psychol. *45*, 5–17.

Ruiz-Mejias, M., Martinez de Lagran, M., Mattia, M., Castano-Prat, P., Perez-Mendez, L., Ciria-Suarez, L., Gener, T., Sancristobal, B., García-Ojalvo, J., Gruart, A., et al. (2016). Overexpression of Dyrk1A, a Down syndrome candidate, decreases excitability and impairs gamma oscillations in the prefrontal cortex. J. Neurosci. *36*, 3648–3659.

Sanderson, D.J., and Bannerman, D.M. (2012). The role of habituation in hippocampus-dependent spatial working memory tasks: evidence from GluA1 AMPA receptor subunit knockout mice. Hippocampus *22*, 981–994.

Sarnyai, Z., Sibille, E.L., Pavlides, C., Fenster, R.J., McEwen, B.S., and Tóth, M. (2000). Impaired hippocampal-dependent learning and functional abnormalities in the hippocampus in mice lacking serotonin(1A) receptors. Proc. Natl. Acad. Sci. USA *97*, 14731–14736.

Sherman, S.L., Allen, E.G., Bean, L.H., and Freeman, S.B. (2007). Epidemiology of Down syndrome. Ment. Retard. Dev. Disabil. Res. Rev. *13*, 221–227.

Siapas, A.G., Lubenov, E.V., and Wilson, M.A. (2005). Prefrontal phase locking to hippocampal theta oscillations. Neuron *46*, 141–151.

Sirota, A., Montgomery, S., Fujisawa, S., Isomura, Y., Zugaro, M., and Buzsáki, G. (2008). Entrainment of neocortical neurons and gamma oscillations by the hippocampal theta rhythm. Neuron *60*, 683–697.

Souchet, B., Guedj, F., Sahún, I., Duchon, A., Daubigney, F., Badel, A., Yanagawa, Y., Barallobre, M.J., Dierssen, M., Yu, E., et al. (2014). Excitation/inhibition balance and learning are modified by Dyrk1a gene dosage. Neurobiol. Dis. *69*, 65–75.

Tejedor, F.J., and Hämmerle, B. (2011). MNB/DYRK1A as a multiple regulator of neuronal development. FEBS J. *278*, 223–235.

Thierry, A.M., Gioanni, Y., Dégénétais, E., and Glowinski, J. (2000). Hippocampo-prefrontal cortex pathway: anatomical and electrophysiological characteristics. Hippocampus *10*, 411–419.

Tort, A.B.L., Kramer, M.A., Thorn, C., Gibson, D.J., Kubota, Y., Graybiel, A.M., and Kopell, N.J. (2008). Dynamic cross-frequency couplings of local field potential oscillations in rat striatum and hippocampus during performance of a T-maze task. Proc. Natl. Acad. Sci. USA *105*, 20517–20522.

Tort, A.B.L., Komorowski, R.W., Manns, J.R., Kopell, N.J., and Eichenbaum, H. (2009). Theta-gamma coupling increases during the learning of item-context associations. Proc. Natl. Acad. Sci. USA *106*, 20942–20947.

Tort, A.B.L., Komorowski, R., Eichenbaum, H., and Kopell, N. (2010). Measuring phase-amplitude coupling between neuronal oscillations of different frequencies. J. Neurophysiol. *104*, 1195–1210.

Varela, C., Kumar, S., Yang, J.Y., and Wilson, M.A. (2014). Anatomical substrates for direct interactions between hippocampus, medial prefrontal cortex, and the thalamic nucleus reuniens. Brain Struct. Funct. *219*, 911–929.

Vega, J.N., Hohman, T.J., Pryweller, J.R., Dykens, E.M., and Thornton-Wells, T.A. (2015). Resting-state functional connectivity in individuals with down syndrome and williams syndrome compared with typically developing controls. Brain Connect. *5*, 461–475.

Vicari, S., Bellucci, S., and Carlesimo, G.A. (2000). Implicit and explicit memory: a functional dissociation in persons with Down syndrome. Neuropsychologia *38*, 240–251.

Wenk, G.L. (2001). Assessment of spatial memory using the T maze. Curr. Protoc. Neurosci *4*, 8.5B.1–8.5A.7.

Winson, J. (1978). Loss of hippocampal theta rhythm results in spatial memory deficit in the rat. Science *201*, 160–163.

Wu, J., and Morris, J.K. (2013). Trends in maternal age distribution and the live birth prevalence of Down's syndrome in England and Wales: 1938-2010. Eur. J. Hum. Genet. *21*, 943–947.

Young, C.K., and McNaughton, N. (2009). Coupling of theta oscillations between anterior and posterior midline cortex and with the hippocampus in freely behaving rats. Cereb. Cortex *19*, 24–40.

Yu, T., Li, Z., Jia, Z., Clapcote, S.J., Liu, C., Li, S., Asrar, S., Pao, A., Chen, R., Fan, N., et al. (2010a). A mouse model of Down syndrome trisomic for all human chromosome 21 syntenic regions. Hum. Mol. Genet. *19*, 2780–2791.

Yu, T., Liu, C., Belichenko, P., Clapcote, S.J., Li, S., Pao, A., Kleschevnikov, A., Bechard, A.R., Asrar, S., Chen, R., et al. (2010b). Effects of individual segmental trisomies of human chromosome 21 syntenic regions on hippocampal long-term potentiation and cognitive behaviors in mice. Brain Res. *1366*, 162–171.

Zhong, Y.M., Yukie, M., and Rockland, K.S. (2006). Distinctive morphology of hippocampal CA1 terminations in orbital and medial frontal cortex in macaque monkeys. Exp. Brain Res. *169*, 549–553.

STAR★METHODS

KEY RESOURCES TABLE

REAGENT or RESOURCE	SOURCE	IDENTIFIER
Chemicals, Peptides, and Recombinant Proteins		
Cresyl Violet acetate	Sigma-Aldrich	C5042
Experimental Models: Organisms/Strains		
Dp1Tyb (Dp(16Lipi-Zbtb21)1TybEm*cf.*)	This paper	NA
Dp10Yey (Dp(10Prmt2-Pdxk)1Yey)	This paper	NA
Dp17Yey (Dp(17Abcg1-Rrp1b)1Yey)	This paper	NA
Dp1Tyb*Dyrk1aKO	This paper	NA
Software and Algorithms		
LWDAQ Software	Open Source Instruments, Brandeis, Boston, USA	http://alignment.hep.brandeis.edu/Software/
Custom MATLAB scripts	This paper	NA
SPSS 24	Statistical Product and Service Solutions, IBM	https://www.ibm.com/analytics/spss-statistics-software
Prism	Graphpad	https://www.graphpad.com/scientific-software/prism/
ImageJ	US National Institutes of Health	https://imagej.nih.gov/ij/
ICY	BioImage Analysis Lab, Institut Pasteur	http://icy.bioimageanalysis.org/

LEAD CONTACT AND MATERIALS AVAILABILITY

Further information and requests for resources and reagents should be directed to and will be fulfilled by the Lead Contact, Matthew Walker (m.walker@ucl.ac.uk). This study did not generate new unique reagents.

EXPERIMENTAL MODEL AND SUBJECT DETAILS

We examined four mouse strains with the following alleles, previously described in Lana-Elola et al. (2016) and Yu et al. (2010a): C57BL/6J.129P2-Dp(16Lipi-Zbtb21)1TybEm*cf.*/Nimr (hereafter referred to as Dp1Tyb); B6;129S7-Dp(10Prmt2-Pdxk)2Yey/J (hereafter referred to as Dp10Yey); B6.129S7 Dp(17Abcg1-Rrp1b)1Yey (hereafter referred to as Dp17Yey) and B6.129P2-*Dyrk1a*^{tm1Mla}. Dp1Tyb, Dp10Yey, Dp17Yey animals were maintained within a facility at University College London, whereas mice for the Dp1Tyb x *Dyrk1a*$^{tm1Mla/+}$ intercross were bred at the Francis Crick Institute, to generate Dp1Tyb*Dyrk1aKO mice in which both alleles were on the same chromosome following a genetic crossover. All strains were maintained in separate colonies as hemizygous mutants backcrossed for over ten generations to C57BL/6J, with age- matched WT littermates used as controls. All experiments were undertaken using male animals, blind to genotype, which was decoded after experimental analysis and reconfirmed using an independent DNA sample isolated from post-mortem tail.

All experiments were performed in accordance with the United Kingdom Animal (Scientific Procedures) Act 1986. Reporting is based on the ARRIVE Guidelines for Reporting Animal Research developed by the National Centre for Replacement, Refinement and Reduction of Animals in Research, London, United Kingdom. Mice were housed in controlled conditions in accordance with guidance issued by the Medical Research Council in Responsibility in the Use of Animals for Medical Research (1993) and all experiments were carried out under License from the UK Home Office and with Local Ethical Review panel approval. Mice were housed in individually ventilated cages (IVC) of 2-5 age-matched animals under controlled environmental conditions (24–25°C; 50%–60% humidity; 12 h light/dark cycle) with free access to food and water.

METHOD DETAILS

Surgical Preparation and Transmitter Implantation for Long-term Recording

Mice were anaesthetised with 2.5%–3% isoflurane (Abbot, AbbVie Ltd., Maidenhead, UK) in 100% oxygen (flow rate of 1-1.5 l/min) via gas anesthesia mask (Model 906, David Kopf Instruments Tujunga, CA, USA) from a recently calibrated vaporizer (Harvard Apparatus, Cambridge, MA). Body temperature was maintained with a heat blanket during surgery. A transmitter (A3028A, Open Source

Instruments, Brandeis, Boston, USA; Chang et al., 2011) was implanted subcutaneously with the depth recording electrodes (a 125 μm diameter teflon-insulated stainless steel electrode with 10kOhm impedance, Open Source Instruments, Brandeis, Boston, USA) positioned in mPFC (1.8 mm anterior, 0.4 mm lateral, 1.5 mm ventral) and dorsal hippocampus (1.85 mm posterior, 1.25 mm lateral, 1.45 mm ventral; Paxinos, 2012). The reference electrode was implanted over the cerebellum posterior to lambda. The whole assembly was held in place with dental cement (Simplex Rapid, Acrylic Denture Polymer, UK). Due to the relatively large diameter of the recording electrode, and prolonged recording period, it is difficult to specify the precise region of dorsal hippocampus from which recordings were made; it is likely that our measurements reflect field potentials summated over a relatively large region. Nonetheless, we estimate that ~57% of recordings were made from CA1 stratum radiatum, ~9% from CA1 stratum oriens, and ~34% from dentate gyrus. A subcutaneous injection of bupivacaine and metacam was provided for post-surgical pain management. At the end of surgery, enrofloxacin (5mg/kg, Baytril, Bayer health care) and pre-warm saline (0.5-1 ml) were administered subcutaneously. The animals were placed in a temperature controlled (25°C) recovery chamber until ambulatory and closely monitored at least 1-2 hours before returning to their home cage to allow recovery for at least 14 days after surgery.

The transmitter, which has no adverse effects (Chang et al., 2016), was chronically implanted for longitudinal data recordings. During all recording sessions, continuous LFP recordings were recorded (bandpass filter: 0.2 Hz to 160 Hz, 512Hz sampling rate with 16 bit resolution) using LWDAQ Software (Open Source Instruments, Brandeis, Boston, USA). Animals were carefully monitored daily and were euthanized at the end of experiment with pentobarbital (25 mg/kg).

Behavioral Testing: T-maze Spontaneous Alternation

Cognitive function in male mice from each strain and associated age-matched WT controls was assessed using the spontaneous alternation paradigm in an enclosed T-maze apparatus (Deacon and Rawlins, 2006). The spontaneous T-maze protocol was chosen because it is powerful enough to interrogate both cognitive and motor function and correlate behavior with changes in neural dynamics, while retaining a relatively simple design. Additionally, this protocol provides clearly defined endpoints to facilitate data analysis, allowing us to simultaneously examine working memory function and differences in movement within a single paradigm, and associate differences in behavior with alterations in neural circuitry across the hippocampus and mPFC. Furthermore, the T-maze is less stressful than other memory tests, such as the Morris water maze or Barnes maze (Harrison et al., 2009), and permits EEG recording.

Animals were transferred to the testing room for 1-2 hours before each experiment to habituate to the environment and achieve an optimal state of arousal. Each mouse was then subjected to ~10 trials per session, and sessions were completed at 3, 6, and 9 months of age (see Tables S1 and S3 for average trial numbers in each group).

During each trial, the animal was first placed in the start chamber for 100 s while reference phase LFP was recorded. Next, the guillotine door separating the start chamber from the central arm was raised and the mouse was allowed to run and choose a goal arm. After making a choice, the guillotine doors separating the central arm from each goal arm were slowly lowered, such that the animal was confined in the chosen goal arm which it could then explore for 30 s. Next, the animal was transferred back to the start chamber, the guillotine door separating the central arm from the goal arms was raised and, after another 100 s delay period in the start chamber, the guillotine door separating the start chamber from the central arm was raised again to allow the mouse a choice between the two open goal arms. Importantly, each trial included a free choice of goal arms on both the sample run and choice run (Figures 1A, S1A, and S1B).

Trials were marked as successful if the mouse chose different goal arms on each run, and failures if the mouse chose the same goal arm on both runs. Alternation rate was defined as the total proportion of successful trials for each animal during each session. Trial latency was calculated as the time between the door isolating the start chamber being raised and the time at which the animals nose reached the decision point (i.e., exiting the central arm of the T-maze) immediately prior to the whole body completely entering the goal arm (indicative of a choice being made; see Figure 1B). This ensures that 'vicarious trial and error' behavior, in which animals approach the decision point and look along either choice arm prior to making a decision, is excluded. Trial data was discarded if the latency on either run exceeded 120 s.

Movement statistics were extracted from video data that covered the central arm, decision point, and initial stages of each goal arm, sampled at a rate of 25Hz, using the single mouse tracker plugin for Icy in ImageJ (de Chaumont et al., 2012). Running speed values were smoothed with a box car filter of 400ms width, and periods of immobility were defined as time frames when the animal's movement speed was lower than 2cm/s.

Histology

At the end of the experiment, the brain was removed and immediately immersed in 4% paraformaldehyde for > 24 hours before being transferred to 30% sucrose post-fixation solution. Brain sections (40-μm thick thickness) were cut using a microtome (Leica SM2000R, Leica Microsystems ltd., United Kingdom) and stained with cresyl violet to allow histological location of the electrode track. This procedure allowed us to verify recording electrode locations, and LFP data were only included in the study if electrode tips were located in mPFC and dorsal hippocampus. In total, LFP data from just one animal was excluded because the recording site was outside the target region (Figure S5).

EEG Data Analysis

LFP Pre-processing

For our initial analyses, continuous LFP recordings from each region were segmented into 10 s epochs that lasted from 5 s before to 5 s after animals reached the decision point on each run (plus 1 s padding, subsequently discarded to account for potential edge effects). Each epoch was visually inspected for artifacts prior to further analysis using custom written MATLAB (Mathworks, Natick MA) code (see Tables S1 and S3 for trial numbers across strains). Trial latency and alternation rate data from trials excluded due to LFP artifacts were nonetheless included in behavioral analyses.

For subsequent analyses in which the relationship between movement statistics and EEG features were examined, continuous LFP recordings from each region were segmented to match the available movement data (plus 1 s padding, subsequently discarded to account for potential edge effects). Any epochs that exhibited artifacts during visual inspection or for which video data (and therefore movement statistics) was either incomplete or unavailable were excluded from subsequent analysis.

Time-frequency Analysis

After de-trending and de-meaning the LFP signal from each trial, time-frequency decomposition was performed using a five cycle complex Morlet wavelet transform, with 1 s of data from the beginning and end of each epoch subsequently discarded to avoid edge effects. Time-frequency representations were then averaged across this time window to provide a power spectrum for each epoch, and each power spectrum was then normalized by its integral to facilitate comparisons between animals. Finally, these normalized power values were averaged across the 6-12Hz theta band to provide an index of theta power in each epoch for statistical comparison.

In addition, to characterize the relationship between theta power and movement, we zero-phase filtered each LFP signal in the 6-12Hz theta band using a 400th order finite impulse response (FIR) filter, discarded 1 s of data from the beginning and end of the signal to avoid edge effects, extracted the analytic signal using the Hilbert transform, and then computed dynamic power and frequency. Running speed data was up-sampled to match the power and frequency time series, allowing us to compute average theta power during movement periods only and to estimate the intercept and slope of the running speed v theta frequency relationship in each animal using linear regression.

Phase-amplitude Coupling Analysis

To assay phase–amplitude coupling in the hippocampal LFP signal, we first computed cross-frequency coherence across a range of phase and amplitude frequencies following Colgin et al. (2009). To do so, we extracted the amplitude at each time point across a frequency range of 20-160Hz from the Morlet wavelet transform described above, and then computed coherence between the original LFP signal and each of these amplitude time series across a phase frequency range of 2-40Hz using a window size of 1 s and an overlap between subsequent windows of 0.5 s. These coherence spectra subsequently index phase-amplitude coupling (PAC) between low frequency phase and high frequency amplitude, and can be aggregated across amplitude frequencies to generate the cross-frequency coherence images shown in Figures 3 and S8.

Visual inspection of cross-frequency coherence images averaged across all animal groups (shown in Figures S8A and S8B) revealed that 6-12Hz theta phase modulated the amplitude of higher frequency oscillations in two distinct bands, 60-120Hz (hereafter referred to as ‘low gamma’, LG) and 140-160Hz (hereafter referred to as ‘high gamma’, HG). We subsequently characterized the magnitude of theta-LG and theta-HG PAC in each epoch by zero-phase filtering the LFP signal separately in the 6-12Hz theta, 60-120Hz LG and 140-160Hz HG bands using a 400th order FIR filter, extracting the analytic signal in each band using the Hilbert transform, and then computing the mean amplitude of the higher frequency oscillations in each of 30 evenly distributed theta phase bins. The resulting vector length of each mean amplitude distribution, computed using the circular statistics toolbox for MATLAB (Berens, 2009), provides an index of theta-LG and theta-HG PAC in each epoch for statistical comparison.

Phase Coupling Analysis

To compute an index of theta phase coherence between LFP recordings from the hippocampus and mPFC in each epoch, we first generated coherence spectra for each epoch using a window size of 1 s and an overlap between subsequent windows of 0.5 s and then averaged coherence values across the 6-12Hz theta range. In addition, to estimate the theta phase lag between concurrent oscillations in these regions, we zero-phase filtered each LFP signal in the 6-12Hz theta band using a 400th order FIR filter, discarded 1 s of data from the beginning and end of the signal to avoid edge effects, extracted the analytic signal using the Hilbert transform, and then computed the circular mean theta phase difference between regions across all time points within each epoch. This provides an indication of the time lag between those signals in the 6-12Hz theta band (computed by dividing the phase difference by the angular frequency at the center of the theta band, i.e., 18π rad/s).

Correcting for Differences in Movement Statistics

Where significant differences in movement statistics between groups existed, we attempted to eliminate any potential confound on concomitant differences in theta coherence and theta-gamma PAC by linear regression. Specifically, we extracted the residual coherence or PAC values after regressing the amount of time spent immobile against those parameters across all animals (mutant and WT), and then assessed the difference in residual values between groups.

QUANTIFICATION AND STATISTICAL ANALYSIS

Detailed statistical analysis was performed using SPSS 24 (Statistical Product and Service Solutions, IBM). All data are presented as mean ± SEM. Comparisons of means were performed using two-tailed Student's t test and one way ANOVA with Tukey post hoc test if the data were normally distributed; Wilcoxon Signed test, Friedman's test, or Mann-Whitney U-test if the data were not normally distributed (with the Shapiro-Wilk test and Kolmogorov-Smirnov test with Lillefors correction used to assess normality of the data distributions). Generalized linear model (GLM) Type III tests followed by Bonferroni post hoc tests were used for analysis of repeated-measures longitudinal data. For circular (i.e., phase lag) data, the Watson-Williams test was used to assess differences between groups (Berens, 2009). Differences were considered statistically significant at $p < 0.05$. For full details of all statistical analyses, please refer to Table S2.

DATA AND CODE AVAILABILITY

All software used in this study is available and listed in the Key Resources Table. This study did not generate any unique datasets or code.

Cell Reports, Volume 30

Supplemental Information

Altered Hippocampal-Prefrontal Neural Dynamics in Mouse Models of Down Syndrome

Pishan Chang, Daniel Bush, Stephanie Schorge, Mark Good, Tara Canonica, Nathanael Shing, Suzanna Noy, Frances K. Wiseman, Neil Burgess, Victor L.J. Tybulewicz, Matthew C. Walker, and Elizabeth M.C. Fisher

Supplemental Information

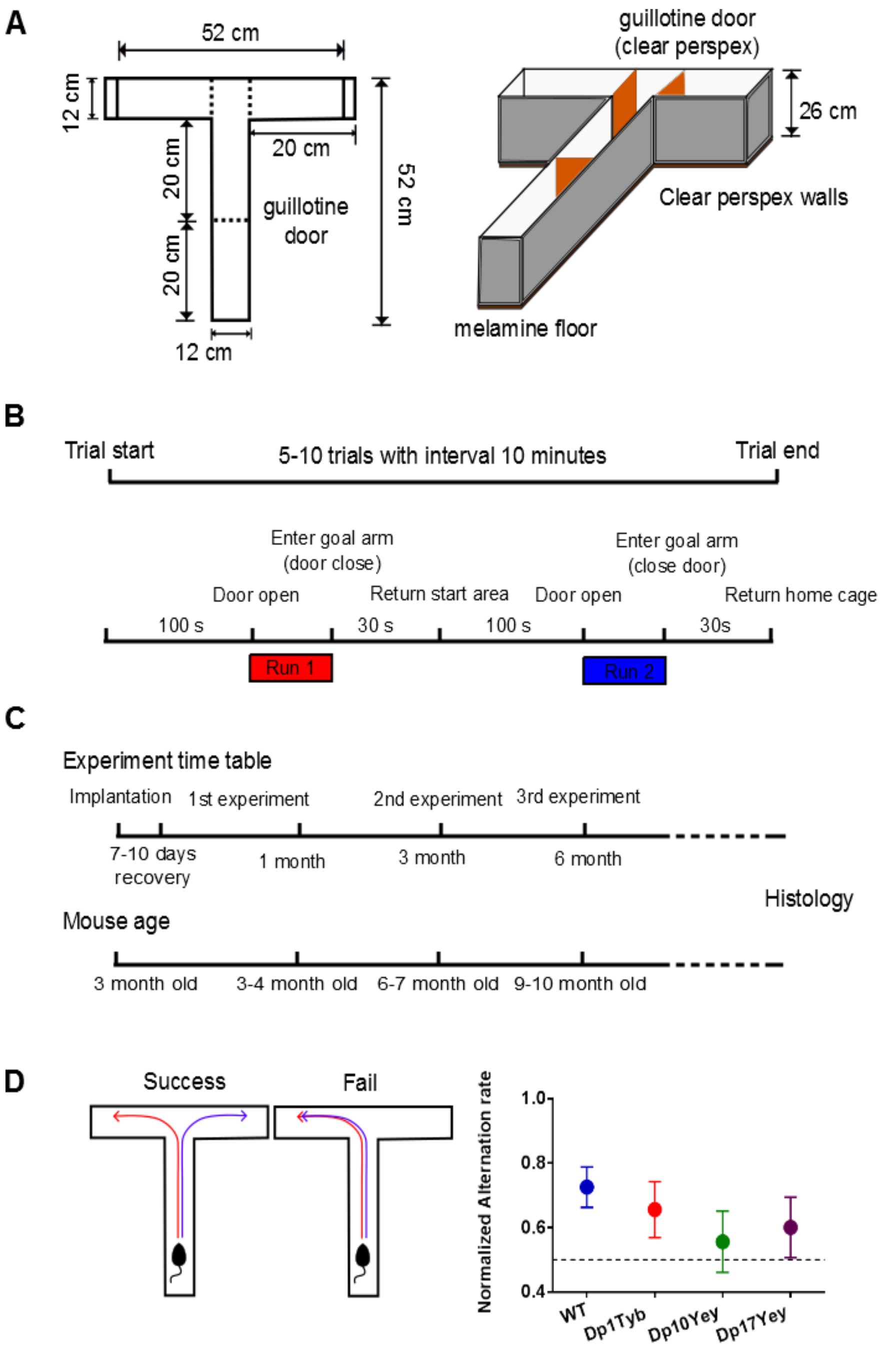

Figure S1: Further Details of the Behavioural Protocol (related to Figure 1)

Experimental protocol for the T-maze spontaneous alternation task, showing: **(A)** schematic of the T-maze; **(B)** trial protocol for probing spontaneous alternation behaviour maze; and **(C)** time line for the longitudinal study. **(D)** Schematic of successful alternation performance (left) alongside mean alternation performance for each mutant mouse group and pooled wild-type (WT) littermates (right, error bars showing 95% confidence intervals). This illustrates that alternation rate in the Dp10Yey animals is significantly different from WT and, importantly, not significantly greater than chance (Z=-1.39, p=0.11). Please refer to Supplemental Table 2 for full details of all statistical analyses.

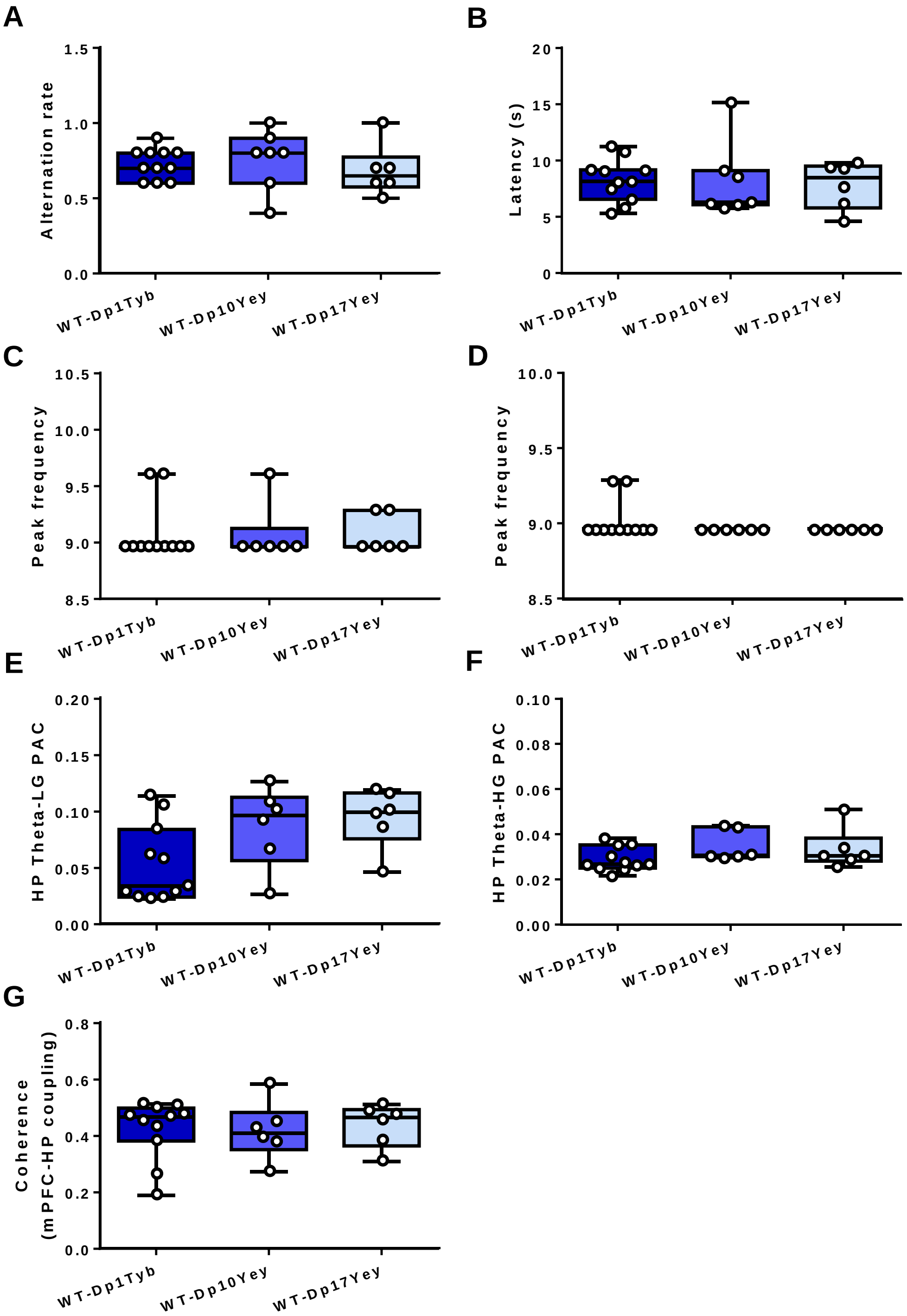

Figure S2: Comparison of Behavioural and Physiological Data across WT Groups (related to Figure 1)

Comparison of **(A)** spatial alternation rate; **(B)** trial latency; peak theta frequency in **(C)** hippocampus and **(D)** medial prefrontal cortex (mPFC); **(E)** theta-low gamma and **(F)** theta-high gamma phase-amplitude coupling in the hippocampus; and **(G)** theta coherence between hippocampus and mPFC across WT cohorts. Data are presented as box-whisker plots indicating the median, 25th and 75th percentiles, minimum and maximum values, with data for individual mice superimposed. There are no significant differences between WT groups in any panel. Please refer to Supplemental Table 2 for full details of all statistical analyses.

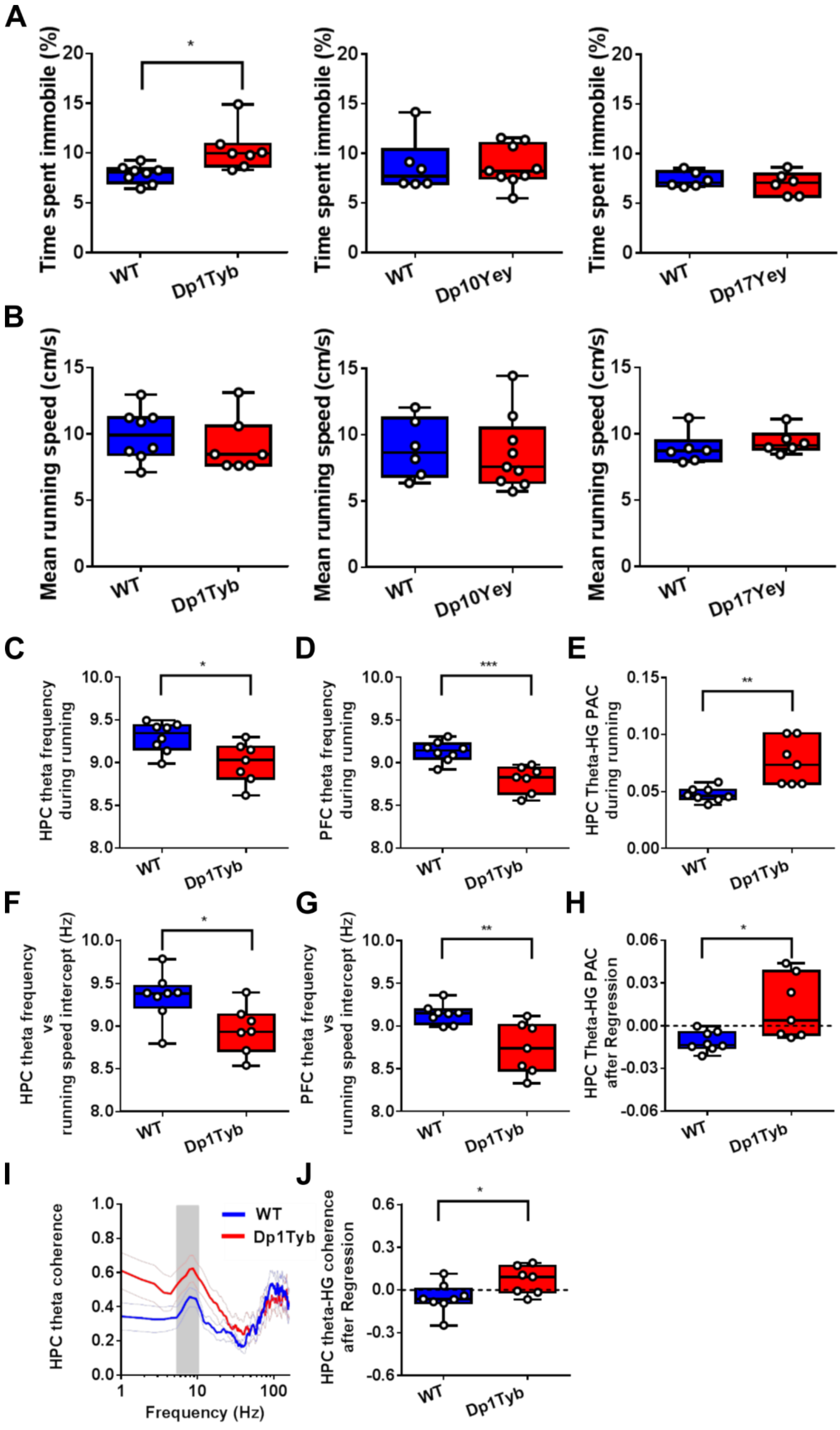

A
Time spent immobile (%)
WT
Dp1Tyb
Dp10Yey
Dp17Yey
B
Mean running speed (cm/s)
C
HPC theta frequency during running
D
PFC theta frequency during running
E
HPC Theta-HG PAC during running
F
HPC theta frequency vs running speed intercept (Hz)
G
PFC theta frequency vs running speed intercept (Hz)
H
HPC Theta-HG PAC after Regression
I
HPC theta coherence
Frequency (Hz)
J
HPC theta-HG coherence after Regression

Figure S3: Comparison of Movement Statistics across Groups (related to Figures 1 and 2)

(A) Relative period of each trial spent immobile (i.e. running speed <2cm/s); and **(B)** mean running speed during movement (i.e. running speed ≥2cm/s) across all mutant mouse and WT control groups, averaged across both runs. These data illustrate that the increased trial latency observed in Dp1Tyb mice results from significantly more time spent immobile ($t(13)=2.46$, $p<0.05$) without any difference in mean running speed during movement ($t(13)=-0.49$, $p=0.63$). No differences in time spent immobile or mean running speed during movement were observed in any other group compared to their WT cohort (all $p>0.38$), or between any of the WT groups (both $p>0.24$). **(C,D)** Average theta frequency and **(F,G)** intercept of the running speed v theta frequency relationship during movement for Dp1Tyb and WT groups in **(C,F)** hippocampus (HPC) and **(D,G)** medial prefrontal cortex (mPFC). Theta frequency during movement is significantly lower in Dp1Tyb animals in both HPC ($t(13)=-2.80$, $p<0.05$) and mPFC ($t(13)=-4.52$, $p<0.001$), due to a reduction in the intercept (HPC: $t(13)=-2.68$, $p<0.05$; mPFC: $t(13)=-3.45$, $p<0.01$) but not the slope (both $p>0.24$, data not shown) of the running speed v theta frequency relationship. **(E)** Average theta-HG PAC in HPC is significantly higher in Dp1Tyb animals when analyses are restricted to movement periods only ($t(13)=3.12$, $p<0.01$). **(H)** Moreover, the influence of average time immobile on theta-HG PAC is removed by linear regression across animals, the difference between groups is still significant ($t(13)=2.88$, $p<0.05$). **(I)** Average theta coherence between HPC and mPFC is significantly higher in Dp1Tyb animals when analyses are restricted to movement periods only ($t(13)=2.44$, $p<0.05$). **(J)** Moreover, if the influence of average time immobile on theta coherence is removed by linear regression across animals, the difference between groups is still significant ($t(13)=2.36$, $p<0.05$). Please refer to Supplemental Table 2 for full details of all statistical analyses.

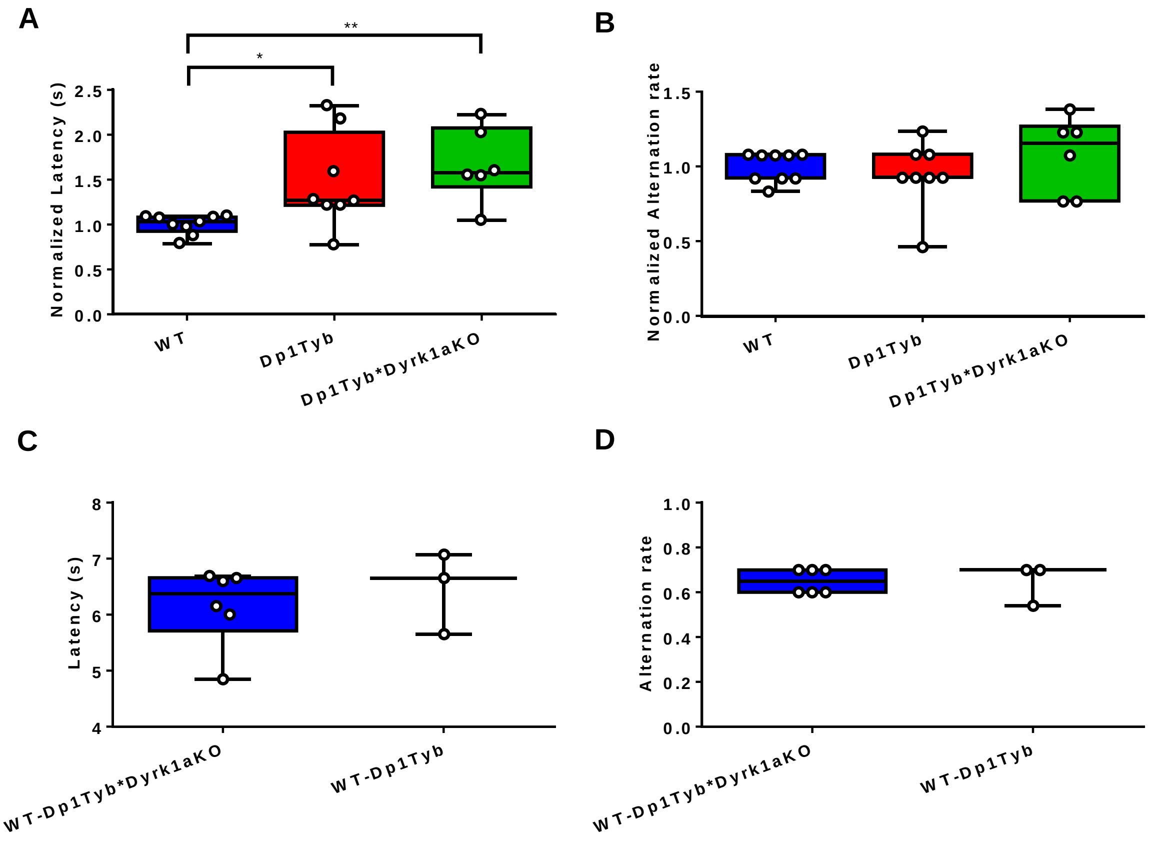

Figure S4: Behavioural Performance in Dp1Tyb and Dp1Tyb*Dyrk1aKO Mice (related to Figure 1)

(A) Trial latency and **(B)** alternation rate averaged over 10 trials for Dp1Tyb and Dp1Tyb*Dyrk1aKO mice compared to their pooled wild-type (WT) control group. All data are normalized to the WT mean. Trial latency differs significantly between groups (One way ANOVA, $F(2,20)=6.21$, $p<0.0001$). Post-hoc Tukey HSD indicates that trial latency is significant longer in Dp1Tyb and Dp1Tyb*Dyrk1aKO mice, compared to pooled WT ($p<0.05$ and $p<0.01$, respectively). **(C)** Comparison of trial latency and **(D)** alternation rate between WT cohorts, illustrating no significant differences in either case (Kruskal-Wallis test, both $p>0.58$). Data are presented as box-whisker plots indicating the median, 25th and 75th percentiles, minimum and maximum values, with data for individual mice superimposed. Please refer to Supplemental Table 2 for full details of all statistical analyses.

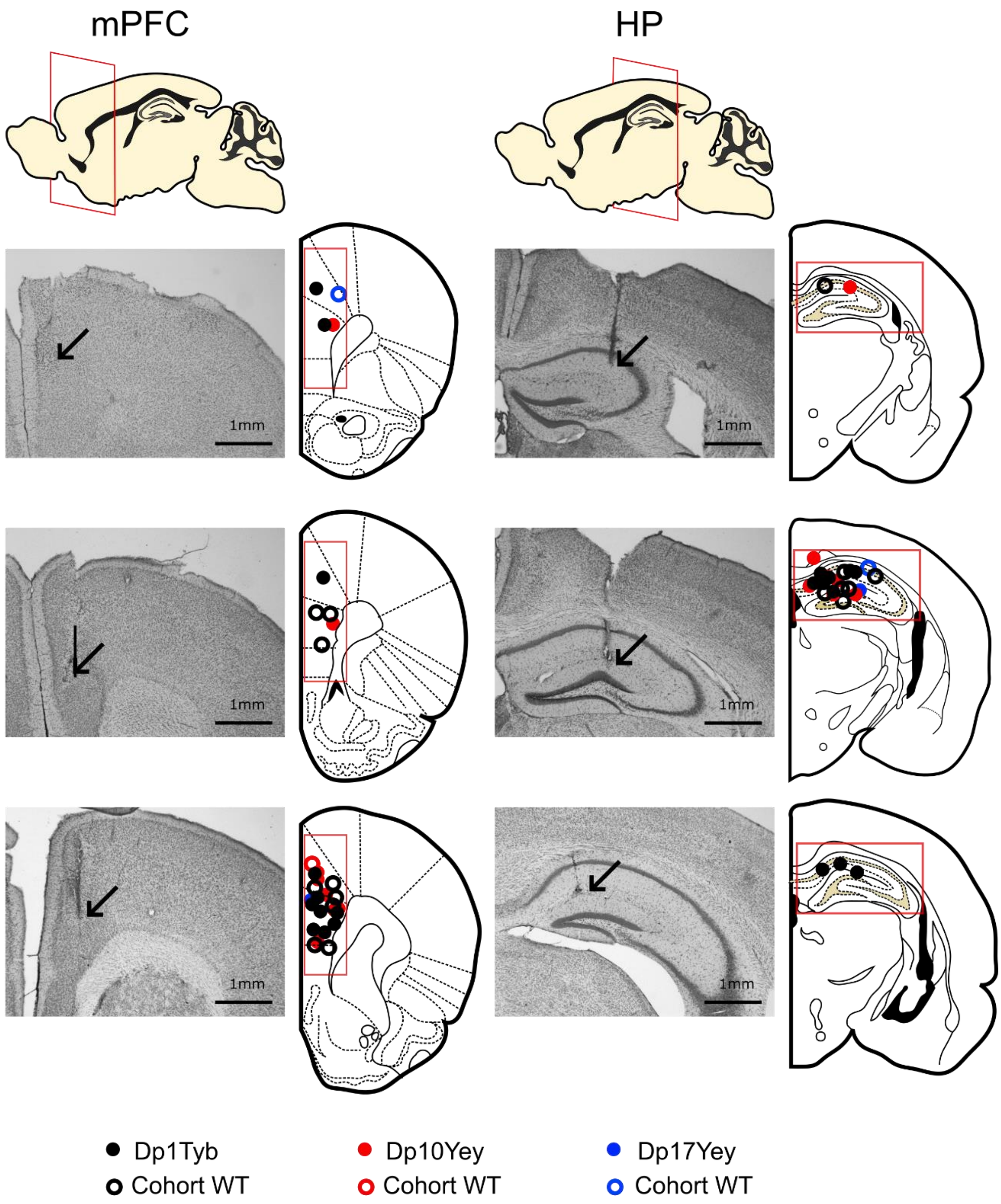

Figure S5: Histology (related to Figure 2)

Coronal sections of Nissl stained brains showing typical locations of recording electrode tips in medial prefrontal cortex and dorsal hippocampus. Arrows indicate the tip of each recording electrode. LFP data were only included in the study if electrode tips were located in mPFC (indicated by coloured rectangle) and dorsal hippocampus (HP, also indicated by coloured rectangle).

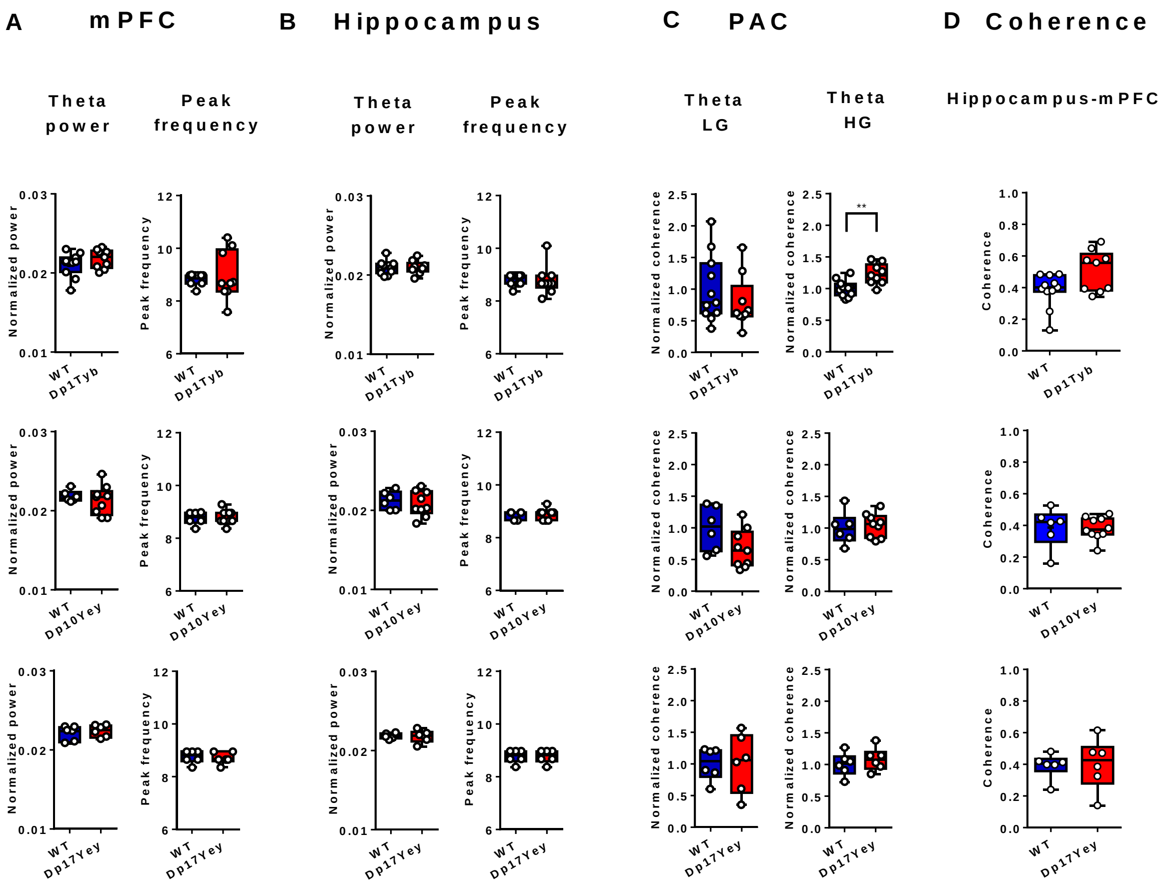

Figure S6: Habituation Phase Analyses (related to Figures 2, 3 and 4)

Comparison of average theta power and peak frequency in the **(A)** mPFC and **(B)** hippocampus, as well as **(C)** theta coherence between regions, across mutant and WT groups. There are no significant differences between any mutant mouse cohort and their WT control group in any of these parameters. Data are presented as box-whisker plots indicating the median, 25th and 75th percentiles, minimum and maximum values, with data for individual mice superimposed. Please refer to Supplemental Table 2 for full details of all statistical analyses.

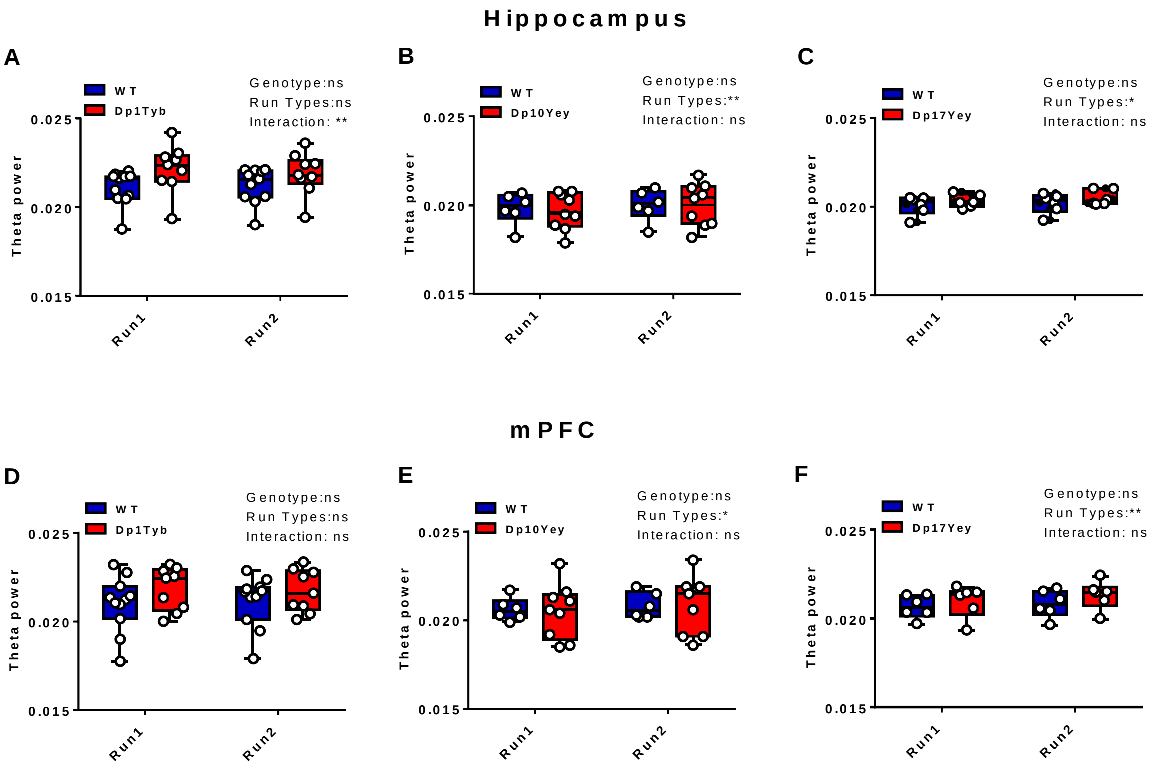

Figure S7: Comparison of Theta Power between Runs (related to Figure 2)

Average theta power during the first (sample) and second (choice) run on the T-maze in the **(A-C)** hippocampus and **(D-F)** mPFC in (A,D) Dp1Tyb and WT; (B,E) Dp10Yey and WT; and (C,F) Dp17Yey and WT animals. Two-way ANOVAs with levels of run (first v second) and genotype (mutant v WT) show a main effect of run on hippocampal theta power in the Dp10Yey and WT ($F(1,14)=6.95$, $p<0.05$), Dp17Yey and WT ($F(1,10)=8.35$, $p<0.05$) cohorts; and a run x genotype interaction in the Dp1Tyb and WT cohort ($F(1,18)=8.86$, $p<0.05$). The same analysis also reveals a main effect of run on mPFC theta power in the Dp10Yey and WT ($F(1,14)=7.67$, $p<0.05$), Dp17Yey and WT ($F(1,10)=17.56$, $p<0.05$) cohorts; but no run x genotype interaction in the Dp1Tyb and WT cohort in this case ($F(1,18)=2.88$, $p>0.05$). Data are presented as box-whisker plots indicating the median, 25th and 75th percentiles, minimum and maximum values, with data for individual mice superimposed. Please refer to Supplemental Table 2 for full details of all statistical analyses.

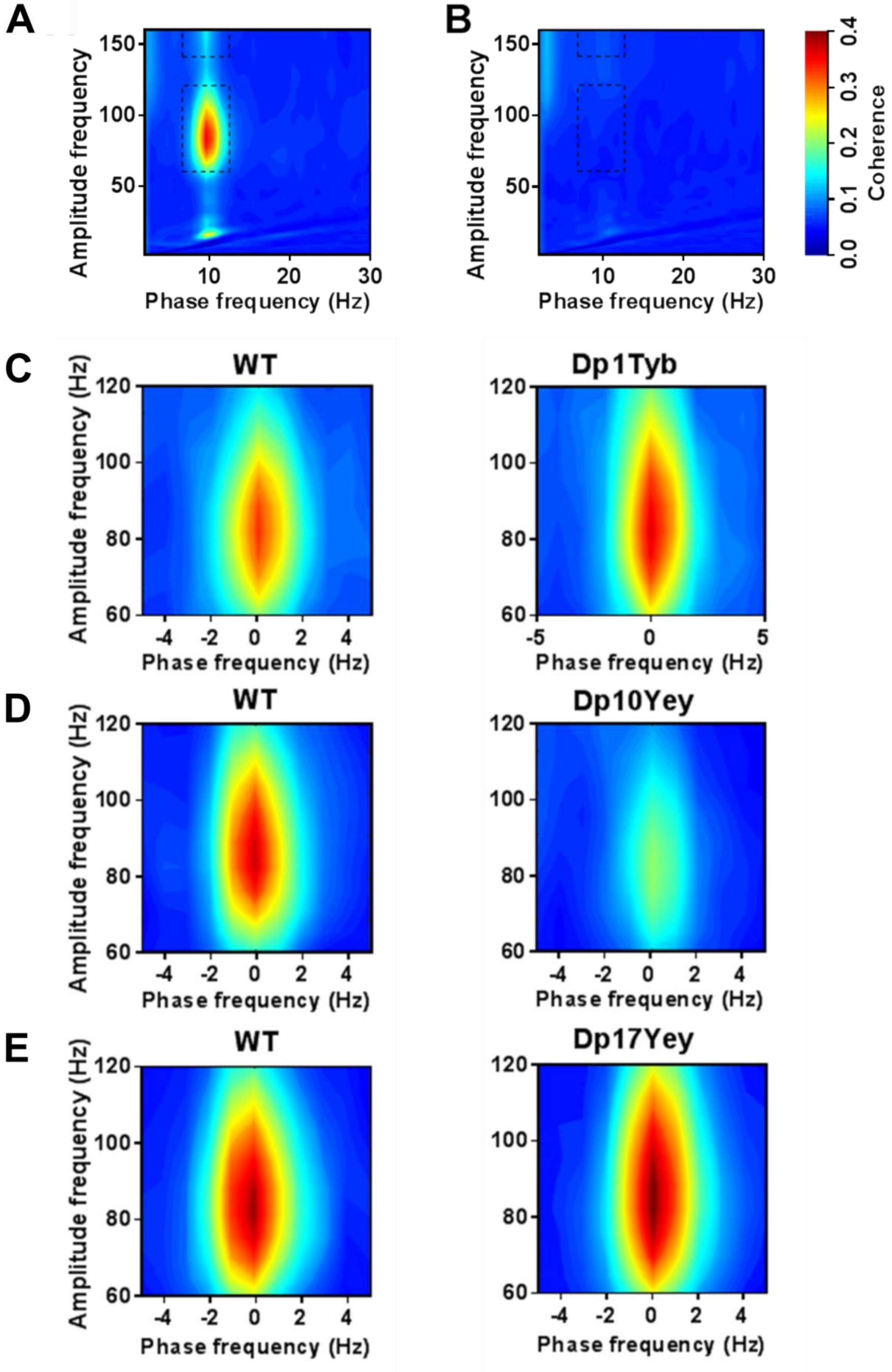

A
B
Amplitude frequency
Phase frequency (Hz)
Coherence
0.0
0.1
0.2
0.3
0.4
C
WT
Dp1Tyb
Amplitude frequency (Hz)
Phase frequency (Hz)
D
WT
Dp10Yey
E
WT
Dp17Yey

Figure S8: Phase-amplitude Comodulograms (related to Figure 3)

(A, B) Phase-amplitude comodulograms, averaged across all DS model and WT control groups at 3 months of age for **(A)** hippocampus and **(B)** mPFC. Visual inspection reveals strong peaks between 6-12Hz theta phase and both 60-120Hz 'low gamma' (LG) and 140-160Hz 'high gamma' (HG) rhythms in the hippocampus, with no strong phase-amplitude coupling apparent in the mPFC (see Figure 3 for details of comparisons between groups). **(C-E)** Comodulograms showing hippocampal phase-amplitude coupling between 6-12Hz theta phase and 60-120Hz 'low gamma' (LG) amplitude separately for the **(C)** Dp1Tyb and WT; **(D)** Dp10Yey and WT; **(E)** Dp17Yey and WT groups. Theta phase frequencies have been aligned to the peak theta frequency in each animal, to facilitate comparison, and colour axes have been matched across groups to illustrate the relative strength of theta-LG phase-amplitude coupling.

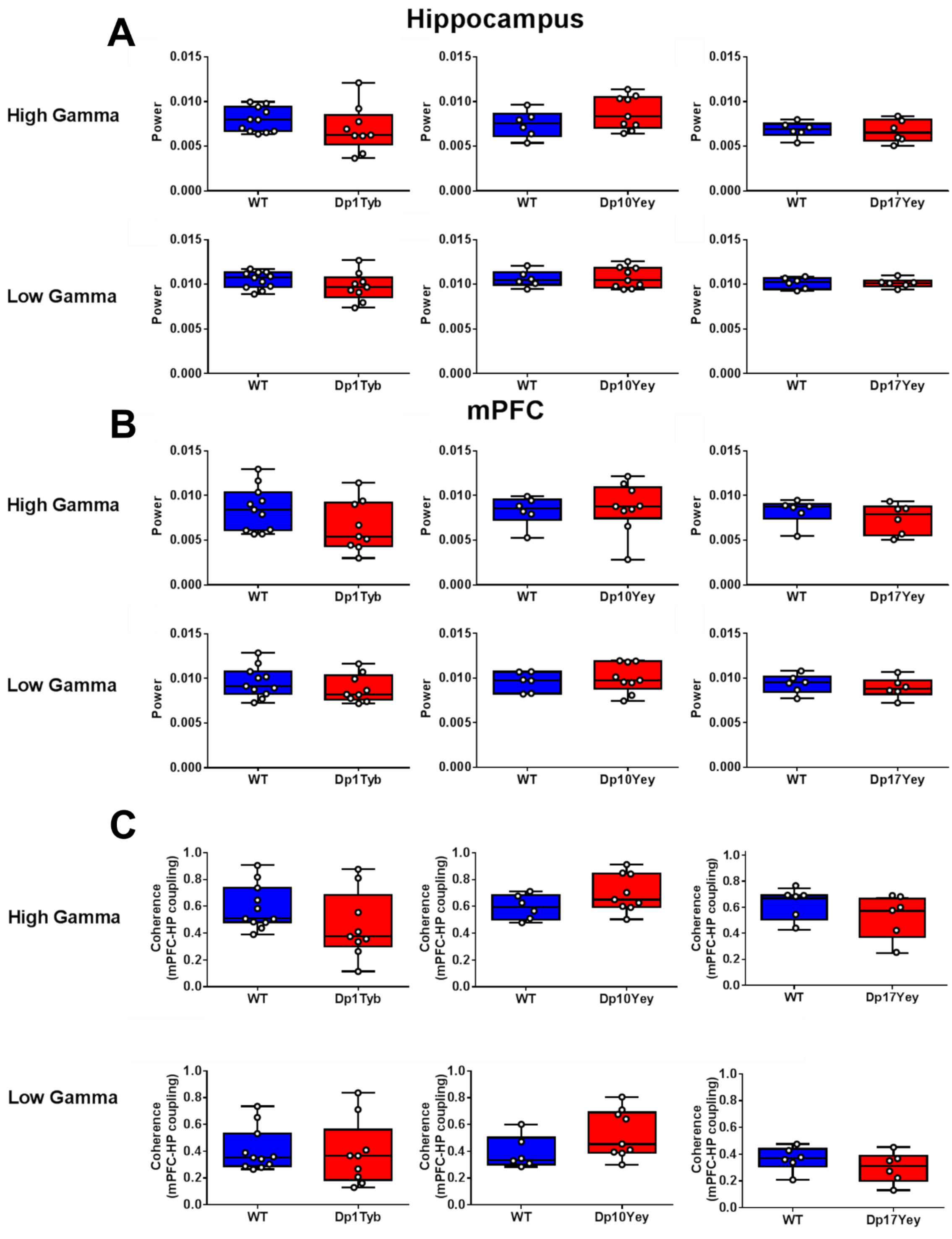

A
Hippocampus
High Gamma
Low Gamma
Power
0.015
0.010
0.005
0.000
WT
Dp1Tyb
Dp10Yey
Dp17Yey
B
mPFC
High Gamma
Low Gamma
C
High Gamma
Low Gamma
Coherence (mPFC-HP coupling)
1.0
0.8
0.6
0.4
0.2
0.0

Figure S9: Gamma Power and Coherence across Groups (related to Figures 2 and 4)

(A,B) Average power in the low (60-120Hz) and high (120-140Hz) gamma bands in **(A)** hippocampus and **(B)** mPFC for each mutant mouse and control group during spontaneous alternation on the T-maze, which show no significant differences in any instance. **(C)** Coherence between the hippocampus (HP) and mPFC in the high- and low- gamma frequency bands for each mutant mouse and WT control group, which show no significant differences in any instance. Data are presented as box-whisker plots indicating the median, 25th and 75th percentiles, minimum and maximum values, with data for individual mice superimposed. Please refer to Supplemental Table 2 for full details of all statistical analyses.

Table S1: Summary of Trial and Animal Numbers at 3 months of Age

Related to Figures 1-4

	Summary						
	**Dp1Tyb**	**WT**	**Dp10Yey**	**WT**	**Dp17Yey**	**WT**	**Mean**
**n**	9	11	10	6	6	6	8
**All Trials (Mean)**	9.33	8.64	8.70	7.83	10.17	9.83	9.08
**All Trials (SD)**	2.00	2.38	2.11	2.79	0.41	0.41	1.68
**Artefact Trials (Mean)**	2.00	1.73	1.80	0.50	1.00	0.33	1.23
**Artefact Trials (SD)**	2.74	2.33	2.66	1.22	0.89	0.82	1.78
**Trials Included (Mean)**	7.33	6.91	6.90	7.33	9.17	9.50	7.86
**Trials Included (SD)**	2.69	2.30	3.63	2.58	0.98	0.84	2.17

Table S3: Summary of Trial and Animal numbers for the Longitudinal Study

Related to Figure 5

3 months							
	Dp1Tyb	WT	Dp10Yey	WT	Dp17Yey	WT	Mean
n	4	6	5	3	6	5	4.83
All Trials (Mean)	8.75	8.17	9.40	6.67	10.17	9.80	8.83
All Trials (SD)	1.89	2.48	0.89	3.51	0.41	0.45	1.61
Artefact Trials (Mean)	2.50	1.17	2.00	0.00	1.00	0.40	1.18
Artefact Trials (SD)	3.32	2.40	3.39	0.00	0.89	0.89	1.82
Included Trials (Mean)	6.25	7.00	7.40	6.67	9.17	9.40	7.65
Included Trials (SD)	2.87	2.61	4.22	3.51	0.98	0.89	2.51

6 months							
	Dp1Tyb	WT	Dp10Yey	WT	Dp17Yey	WT	Mean
n	4	6	5	3	6	5	4.83
All Trials (Mean)	12.50	10.83	10.00	10.33	10.00	10.00	10.61
All Trials (SD)	4.36	1.17	0.00	0.58	0.00	0.00	1.02
Artefact Trials (Mean)	0.00	0.33	0.00	0.00	0.67	0.20	0.20
Artefact Trials (SD)	0.00	0.52	0.00	0.00	0.82	0.45	0.30
Included Trials (Mean)	12.50	10.50	10.00	10.33	9.33	9.80	10.41
Included Trials (SD)	4.36	1.52	0.00	0.58	0.82	0.45	1.29

9 months							
	Dp1Tyb	WT	Dp10Yey	WT	Dp17Yey	WT	Mean
n	3	4	4	2	6	5	4
All Trials (Mean)	10.33	12.25	10.25	10.00	10.17	10.40	10.57
All Trials (SD)	0.58	5.19	2.06	0.00	0.41	0.55	1.46
Artefact Trials (Mean)	0.00	0.25	0.00	0.50	0.33	0.00	0.18
Artefact Trials (SD)	0.00	0.50	0.00	0.71	0.52	0.00	0.29
Included Trials (Mean)	10.33	12.00	10.25	9.50	9.83	10.40	10.39
Included Trials (SD)	0.58	4.69	2.06	0.71	0.75	0.55	1.56
